# Green composite aerogel based on citrus peel/chitosan/bentonite for sustainable removal Cu(II) from water matrices

**DOI:** 10.1038/s41598-023-42409-2

**Published:** 2023-09-18

**Authors:** Jing Nie, Dan Feng, Jiangwei Shang, Bate Nasen, Tong Jiang, Yumeng Liu, Siyi Hou

**Affiliations:** 1 Key Laboratory of Pollutant Chemistry and Environmental Treatment, College of Resources and Environment, Yili Normal University, Yining, 835000 China; 2 College of Chemistry and Chemical Engineering, Yili Normal University, Yining, 835000 China

**Keywords:** Environmental sciences, Materials science

## Abstract

Here, we propose a green and sustainable 3D porous aerogel based on citrus peel (CP), chitosan (CS), and bentonite (BT). This aerogel is prepared through a simple sol–gel and freeze-drying process and is designed for efficient capture of Cu(II) ions from water matrices. CCBA-2, with its abundance of active binding sites, exhibits an impressive Cu(II) adsorption yield of 861.58 mg/g. The adsorption isotherm and kinetics follow the Freundlich and pseudo-second-order models, respectively. In the presence of coexisting mixed-metal ions, CCBA-2 demonstrates a significantly higher selectivity coefficient (K_d_^Cu^ = 1138.5) for removing Cu(II) ions compared to other toxic metal ions. Furthermore, the adsorption of Cu(II) ions by CCBA-2 is not significantly affected by coexisting cations/anions, ionic strength, organic matter, or different water matrices. Dynamic fixed-bed column experiments show that the adsorption capacity of Cu(II) ions reaches 377.4 mg/g, and the Yoon-Nelson model accurately describes the adsorption process and breakthrough curve. Through experiments, FTIR, and XPS analyses, we propose a reasonable binding mechanism between CCBA-2 and metal cations, involving electrostatic attraction and chemical chelation between Cu(II) and the functional groups of the aerogel. CCBA-2 saturated with Cu(II) ions can be successfully regenerated by elution with 1 M HNO_3_, with only a slight decrease in adsorption efficiency (5.3%) after 5 adsorption–desorption cycles. Therefore, CCBA-2 offers a cost-effective and environmentally friendly material that can be considered as a viable alternative for the green and efficient removal of toxic Cu(II) ions from wastewater.

## Introduction

Water contamination with heavy metals is a significant issue that needs to be effectively addressed due to the serious risk it poses to ecosystem sustainability and human health^1,2^. Among various heavy metals, Cu(II) is an essential trace element for the human body. However, exposure to excessive amounts can cause serious health problems such as Menkes, Wilson’s and Alzheimer’s diseases^3–5^. Cu(II) is frequently found in wastewater from various sources, including electrolysis, metal plating, battery disposal, sewage sludge, and catalysts^6–8^. The United States Environmental Protection Agency (USEPA) has set a maximum Cu(II) contamination level of 1.3 mg/L in surface or groundwater supplied as drinking water^9–11^. So far, various techniques have been developed to remove heavy metal pollution from water, including chemical precipitation, ion exchange, electrodeposition, and adsorption^12–15^. Each approach has its drawbacks and limitations^16^. Relatively, the adsorption method is commonly considered as a simple and effective method for treating heavy metal wastewater due to its economic application, simple design and operation, and environmental friendliness^17–19^. However, the successful application of adsorption largely depends on the properties of the adsorbent^20^. To date, researchers have developed diverse adsorbents to remove heavy metal ions from liquid matrices. Activated carbon is a typical traditional adsorbent, but it is considered unsustainable due to the high energy consumption and greenhouse gas emissions involved in its production process^21^. Other adsorbents, such as MOFs, COFs, and clay, are usually in the form of powders or nanoparticles, which makes the separation and reuse steps after treatment costly. In particular, in a fixed-bed column adsorption device, it is challenging to prevent the powder filler from flowing out with the treatment medium. While magnetizing the adsorbent can improve the issue of poor separation^22^, there are still deficiencies, such as unsatisfactory adsorption effect or short magnetic function cycle. Therefore, in the premise of high adsorption capacity, high removal rate with different forms of adsorbent (powder, particle, block, etc.), whether the adsorbent is economic, selective, environmental-friendly and sustainable, whether it is easy to regenerate and reuse, are key indicators to consider in its synthesis process.

Aerogel is a type of bulk material with a highly interpenetrating porous internal structure, which has unique characteristics of ultra-low density, large specific surface area, high adsorption capacity, controllable shape, easy recovery and reutilization^18^. This makes it easy to be separate from an aqueous solution, making it an excellent adsorbent for capturing heavy metal pollutants^23^. However, aerogels that have been developed and popularized so far include graphene^3^, silicon^24^, and carbon nanotubes^25^, which are subject to oil-derived precursors and may produce secondary pollution. Hence, exploring cheap and sustainable building blocks from natural polymers to develop green composite aerogels for coping and mitigating intractable water pollution with toxic metal ions is a path worth exploring^26^. Given this, natural polymers like chitosan, cellulose, cyclodextrin, sodium alginate, starch, and waste biomass^27^, are ideal for preparing green and sustainable composite adsorbents due to their non-toxicity, affordability, and availability, as well as being promising candidates for trapping toxic pollutants based on selectivity in complex water matrices^26^. Usually, producing aerogel adsorbents from natural waste biomass is even more cost-effective and aligns with the reduce-reuse-recycle principles of a circular economy. Recently, aerogels developed from natural waste biomass, such as waste reed^18^, posidonia oceanica waste biomass^28^, grapefruit peel^29^ and bagasse^16^, have been reported for water pollution remediation. However, there is limited research on the fabrication and application of aerogels based on citrus peel (CP) for water pollution remediation.

As one of the most common waste-biomass in daily life, CP is produced at about 1.1–1.2 billion tons per year from its processing industry^30^. In China, CP can be used as an ingredient in Chinese medicine formulations, but the vast majority (99%) of the peel is abandoned as waste^31^, which poses a huge challenge for soil, land, and solid waste management^30^. Therefore, in view of exploring comprehensive utilization of this waste renewable biomass resource, many researchers have worked tirelessly, successfully utilizing it in the pharmaceutical and food industries, as well as in the production of biogas, fuels and ethanol through microbial processes, physicochemical and fermentation^32–34^. Beyond that, as a type of lignocellulosic biomass derived from citrus fruits, CP is mainly composed of cellulose, hemicellulose, lignin, and pectin (galacturonic acid), which contain heavy metal chelating groups such as hydroxyl, methoxy group and carboxyl^35^. Cellulose, as a main ingredient in CP’s constitution, forms strong inter/intramolecular hydroxyl bonding and large Van der Waals forces, making CP exhibit outstanding mechanical strength, revealing unique properties in preparing aerogel for removing heavy metal pollution in wastewater^23^. Furthermore, the price of lignocellulose-based adsorbents is about US $48/ton, which is quite affordable compared to commercial substitutes, e.g., active carbon (US $400–1500/ton)^30^. In this scenario, reasonable recycling of citrus waste is meaningful for reducing environmental pressure and improving water quality in polluted water bodies. However, the abundant hydroxyl group on its surface can easily form hydrogen bond interaction with water molecules, so that the physically cross-linked aerogel is easily depolymerized in liquid^36^. Generally, chemically integrating it with other natural functional polymers could improve it^30^.

Chitosan (CS) is a natural polymer derived from the deacetylation of chitin, which possesses the merit of being abundant, renewable and biocompatible^37^. CS is the only basic polysaccharide in natural polymers, containing a rich free –NH_2_ group. Its neighbor, the –OH group, can effectively chelate bivalent metal ions^9^. However, pure chitosan aerogel has poor mechanical stability and acid resistance^38^. To improve this, CS has been integrated with other polymers such as konjac glucomannan^39^, graphene oxide^40^, orange peel^41^, montmorillonite^17^, silica hybrid^42^ and carboxymethyl cellulose^43^/microcrystalline cellulose^2^. Among these options, lignocellulosic biomass and clay are the cheapest and most easily available. Bentonite (BT), mainly composed of montmorillonite (MMT) with the molecular formula of M_x_(H_2_O)_4_{A1_2−x_Mg_x_) [Si_4_O_10_](OH)_2_}^44^, is widely distributed in Xinjiang Province, China. Due to the presence of abundant hydroxyl groups, large specific surface area, and surface electronegativity, the removal of metal cations by BT have been widely reported^3,17,45,46^. In addition, studies reveal that the mechanical properties of composites can be effectively improved by using well-dispersed clay^47^. However, pure MMT is a powder form, making recover and regenerate after adsorption difficult. The development of MMT aerogel has solved this issue to some extent, but the pure MMT aerogel is suffers from remarkably low adsorption properties^44,46^. Based on this, various binary and ternary composite compounds have been developed, such as CS/MMT aerogels^17^, cellulose/montmorillonite composite aerogel^48^, CS/CNF/MMT^49^, and BT-CS-MCCA^50^, etc. However, to our knowledge, the synthesis of aerogels from CP, CS and BT (CCBA) with a regular three-dimensional porous structure for selectively removing Cu(II) ions in complex water matrices has received less attention. This research aims to develop a green and sustainable adsorbent for water pollution remediation.

In this study, an eco-friendly aerogel, CCBA, was prepared by a simple sol–gel method combined with freeze-drying technology, which was used for efficient and selective removal of Cu(II) ions in liquid. The morphological structure, chemical composition and mechanical properties of CCBA aerogels were briefly characterized and quantified. The study aimed to evaluate the selective adsorption performance of CCBA for Cu(II), and investigate the effects of different experimental conditions on the removal process, such as solution pH, coexisting ions, ionic strength, humic acid and different water matrices. The practicality of the aerogel was explored through fixed-bed column adsorption experiments, and the removal performance of CCBA-2 on target metal ions under different initial concentration conditions was studied. The comprehensive utilization and ecological risk of low concentration effluent were explored through pot experiments. Finally, the adsorption and removal mechanism of CCBA-2 toward Cu(II) was analyzed using FTIR, EDS, and XPS.

## Results and discussion

### Preparation and characterization of CCBA

**Figure 1:**
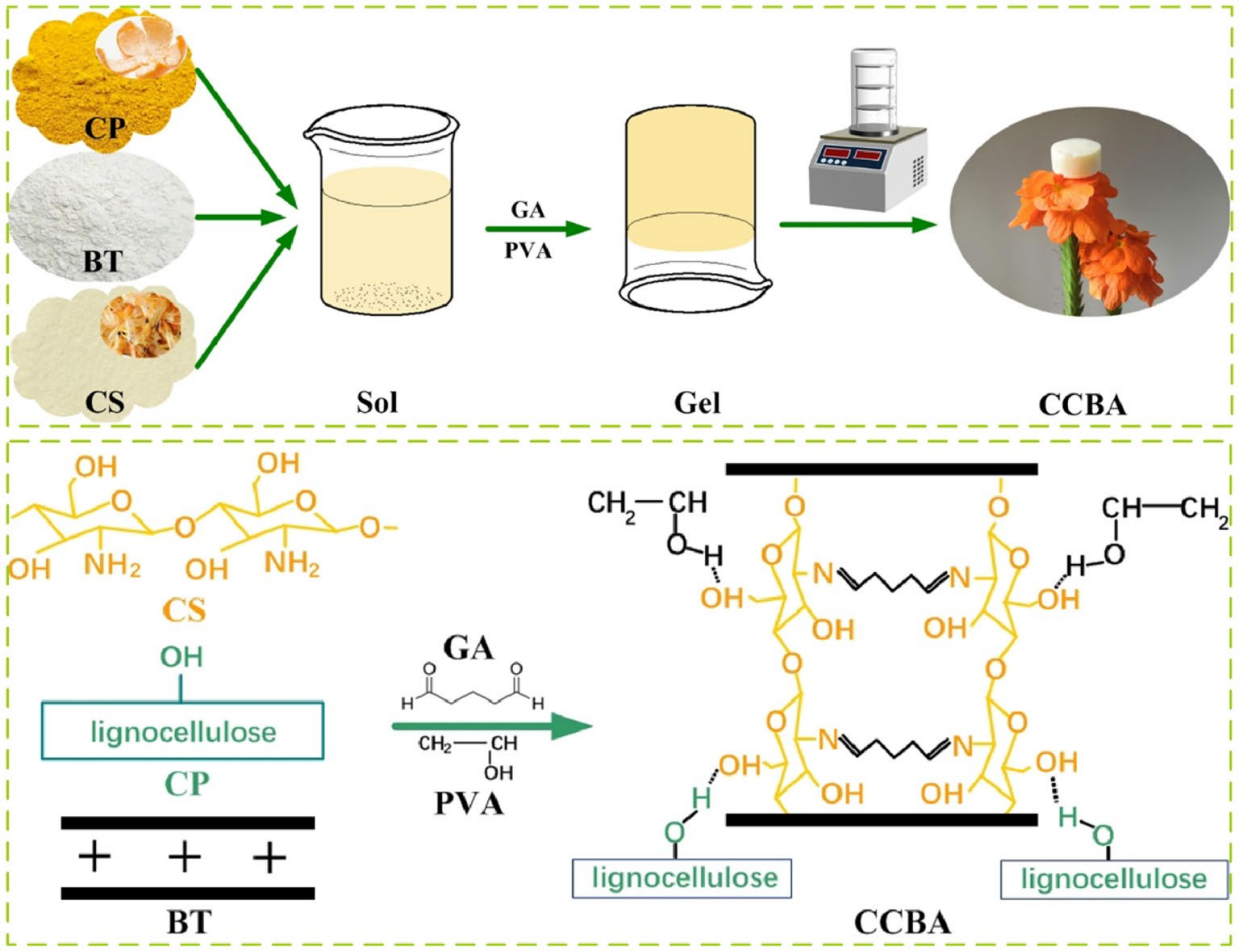
Preparation process and mechanism of CCBA.

Figure 1 illustrates the straightforward process and synthesis mechanism of chemically modified CCBA. Briefly, a stable super-macromolecular network structure is formed through the Schiff base reaction between the amino group of CS and the aldehyde group of glutaraldehyde (GA). The hydroxyl groups on CS, CP, and BT are connected through hydrogen bonds formed by physical cross-linking, further strengthening the macromolecular structural system. Polyvinyl alcohol (PVA) acts as a pore-forming agent, increasing the inner pores inside the aerogels. After freeze-drying and dehydration, CCBA is assembled into an ultra-light spongy aerogel with an interpenetrating three-dimensional network. In the CCBA synthesis process, CP, as a substrate material, makes the aerogel formed by GA and CS more ductile. BT is uniformly dispersed in the entire composite system, optimizing the mechanical properties of the aerogel material. The final preparation process results in CCBA-2 with a density of 0.025 g/cm^3^, which can be supported by a delicate petal.

**Figure 2:**
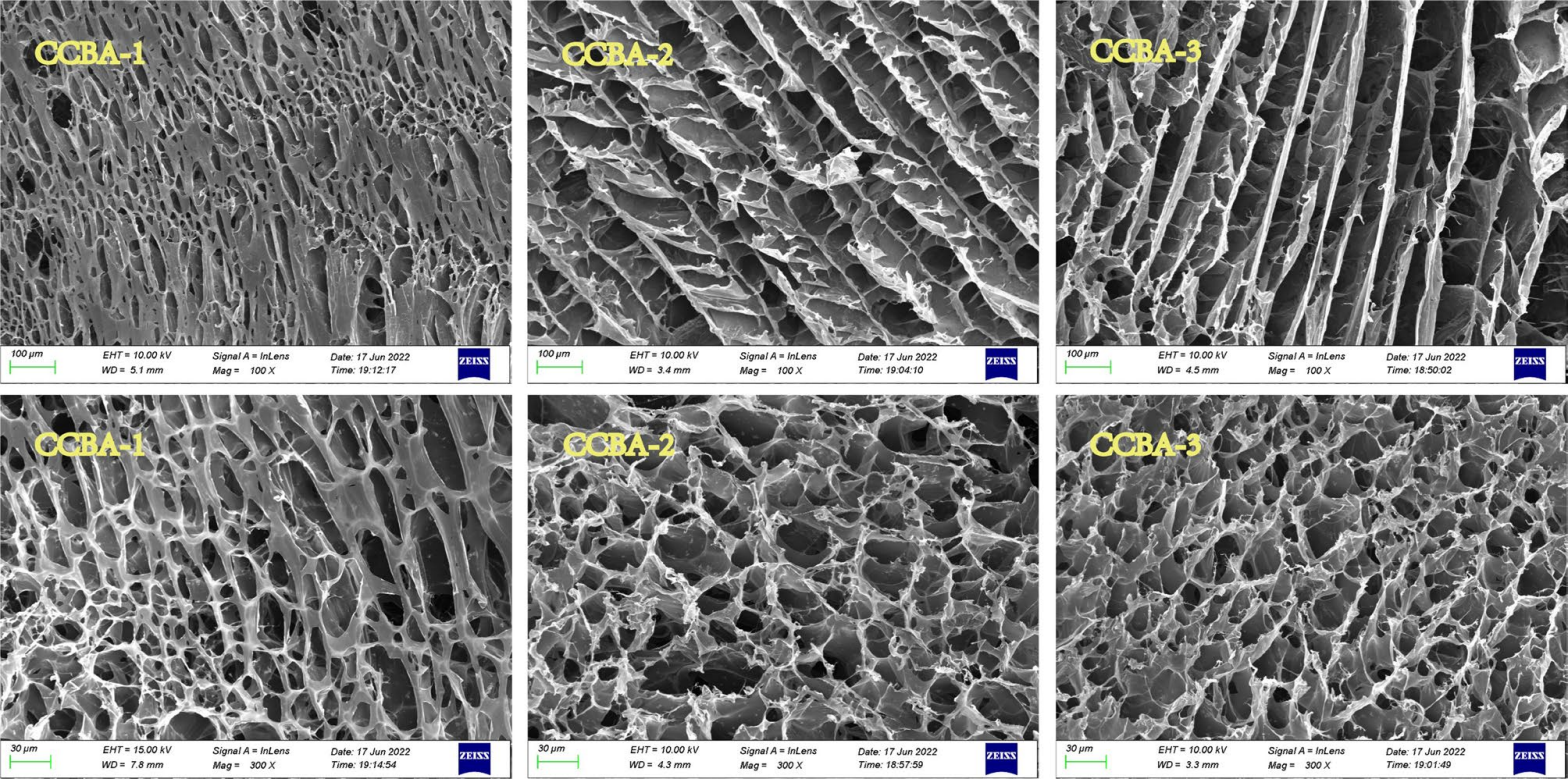
SEM image of three aerogels.

Figure 2 and Supplementary Fig. S1 display the SEM images of CCBA and CSA/CSPA, respectively. Different from the two-dimensional honeycomb or irregular micropore structures exhibited by pure CSA, CSPA aerogel, or previously reported montmorillonite/cellulose aerogel^48^, untreated waste office paper/chitosan aerogel^37^, cellulose and chitosan aerogel^18^, chitosan/montmorillonite composite aerogel^17^, the porous structure in the as-prepared aerogels appears more resilient and orderly. Studies have shown that the addition of BT can increase the density of mesopores and the mechanical strength of aerogels^48^. As seen in Fig. 2, the inner pore density in CCBA increases with the increase in CP content. The slit-shaped pores in CCBA-1 are sturdy, which is different from the relatively fragile pore structure of CCBA-2 and CCBA-3. Even when compared with a similar bentonite modified chitosan/microcrystalline cellulose aerogel^50^, CCBA still exhibits a more ductile, dense and compact three-dimensional porous-structure, facilitating the anchoring of pollutants by active binding sites on CCBA.

**Figure 3:**
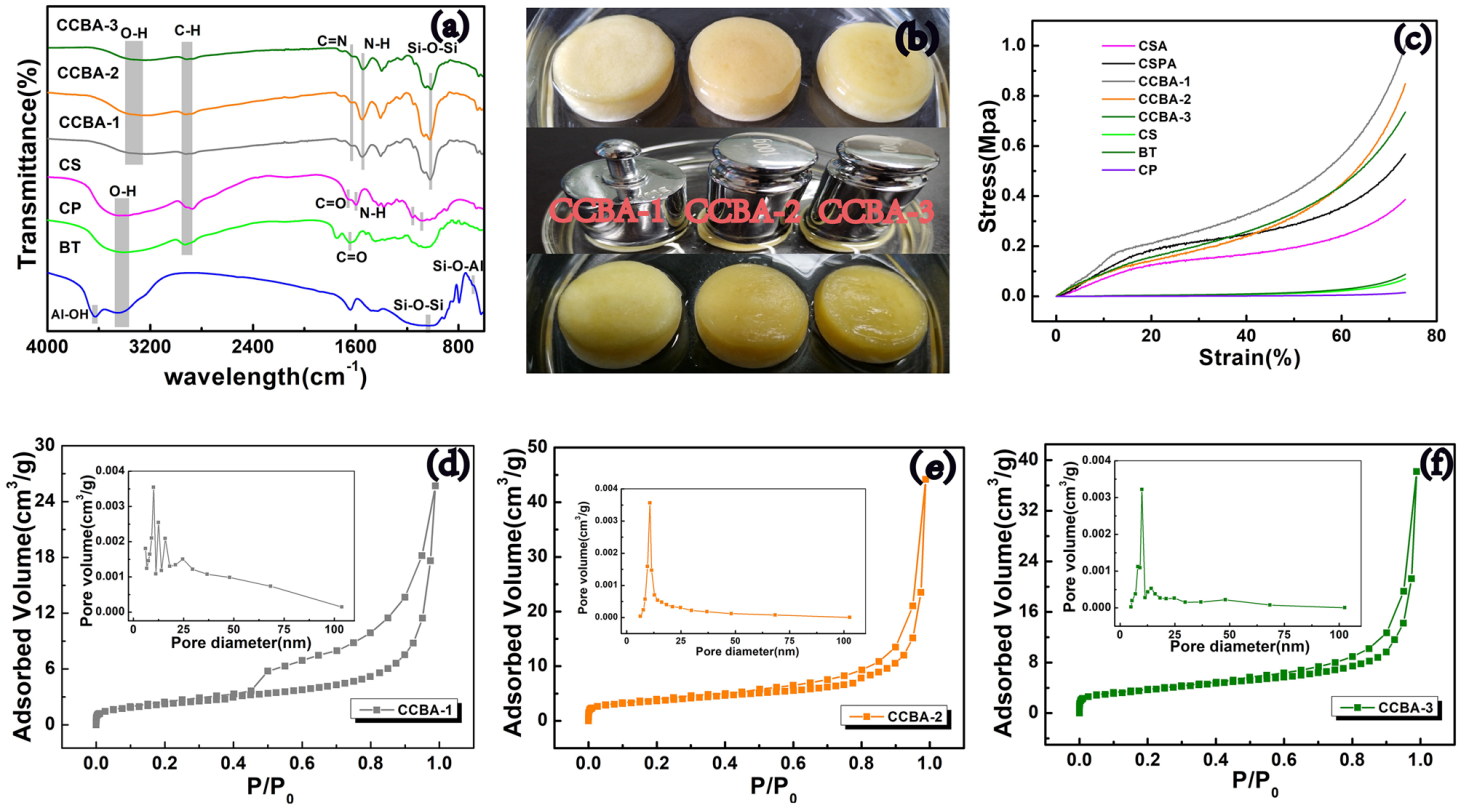
(**a**) FTIR spectrum of CP, CS, BT, CCBA-1, CCBA-2 and CCBA-3, (**b**) digital graph of CCBA before and after compression under water saturated condition, (**c**) strain–stress curve, (**d**–**f**) adsorption–desorption curves and pore diameter distribution of CCBA.

FTIR spectroscopy was used to observe the changes in functional groups during the formation of the aerogel. In Fig. 3a, the sharp peak at 3630 cm^−1^ corresponds to the stretching vibration of Si–OH in BT, followed by a broadband at 3457 cm^−1^ of O–H stretching, indicating the existence of water molecules and intermolecular hydrogen bonding in BT, which acted as a linkage between tetrahedral and octahedral layers^41^. The wideband at 3360–3460 cm^−1^ in pristine CS represents the O–H and N–H stretching vibrations, while the peaks at 1660 and 1600 cm^−1^ are attributed to the C=O group band and N–H bending vibration of amide II^38,51^. The bands at 2871 cm^−1^ and 1082 cm^−1^ correspond to the C–H stretching vibration and C–O skeletal stretching vibration involve in the saccharide structure of CS. For pure CP, the broadband at 3409 cm^−1^ indicates the presence of the O–H functional group due to intramolecular/intermolecular hydrogen bonding. The bands at 2925 and 1640 cm^−1^ are related to the C–H and C=O stretching vibrations of carboxy groups in CP compounds such as cellulose, lignin, and pectin^41^.

For CCBA aerogels, the O–H/N–H stretch vibrations at 3360–3460 cm^−1^ of CS, 3409 cm^−1^ of CP and 3457 cm^−1^ of BT all shift to the vicinity of 3325 cm^−1^, and the strength of these bands is obviously weaker than the raw materials, suggesting their involvement in the grafting or blending mechanism of the aerogel synthesis process. The formation of a fresh C=N bond at 1633 cm^−1^ confirms the successful chemical cross-linking process through the Schiff's base reaction between GA and CS^38,41^. The free radical polymerization mechanism between the O–H and C–H groups in the composite system removes water molecule, resulting in the weakening of the intensity of both peaks compared to pristine BT, CP and CS. The peak at 1600 cm^−1^ (amide II) in CS shifts to around 1555 cm^−1^, indicating CS's participation in the chemical reaction during the adsorbent preparation. The disappearance of the characteristic peak at 3630 cm^−1^ of Al–OH in BT suggests its involvement in the synthesis reaction. Although the characteristic peak of BT is not obvious in CCBA, different proportions of BT in each aerogel can be clearly seen from the EDS spectrum (Supplementary Fig. S2).

Furthermore, the stability and mechanical properties of the adsorbent play an important role in its practical applications, which are determined by many factors, such as the preparation method, the ratio of each component, the type and microstructure/macrostructure of the raw material. Figure 3b and Supplementary Video 1 in supporting information (SI) show the CCBA aerogels before and after compression under water saturated conditions. Supplementary Figure S3 presents a digital graph of CSA and CSPA after loading the same weight (500 g) under water saturated conditions. Macroscopic cracks appear on the surface of the CSA after compression, and CSPA also exhibits certain fissures, making them inadequate for practical regeneration and reuse. In contrast, CCBA does not crack after compression. CCBA-1 can almost fully recover after compression, followed by CCBA-2, while CCBA-3 recovers about 70% of its original height. The stress–strain (σ–ε) measurements process explains this phenomenon. As shown in Fig. 3c, the mechanical property of CCBA is significantly enhanced compared to pure CP, CS, BT, or modified CSA and CSPA. In the three as-prepared aerogels, the stress of the aerogel increases significantly with the increase/decrease of BT/CP content in the CCBA system. The stress reaches the maximum value (1.0 MPa) at strain of 73.4% when the mass ratio of BT/CP is 2:1 (CCBA-1). Young’s modulus for BT, CS, CP, CSA, CSPA, CCBA-1, CCBA-2 and CCBA-3 of 118.95, 95.16, 20.23, 525.89, 773.35, 1366.49, 1154.66 and 1000.95 kPa, respectively. This further proves the superior mechanical stability of CCBA compared to crude substrate, CSA, or CSPA. The addition of rational dose of BT in the composite system optimizes the mechanical properties of the inner-pore structure^47,48^, while CP makes the adsorbent more resilient and resistant to cracking under pressure. The synergistic effect of CP and BT greatly improves the mechanical properties of CCBA. Besides, the hydrophilicity of the material is also an important index to evaluate the practicality of the aerogel. Supplementary Video 2 in the supporting information (SI) shows that water immediately spreads and penetrates into the aerogel when dropped on its surface, indicating that CCBA has good hydrophilicity to effectively adsorb metal ions in aqueous solution.

The N_2_ adsorption isotherms of CCBA were measured to determine the pore size distribution and adsorption–desorption curve. Figure 3d–f and Supplementary Table S1 illustrate the corresponding results. The three samples exhibit a type-IV curve and an H3 hysteresis loop, indicating the presence of mesopores in the aerogels^3^. CCBA-1 has a wider hysteresis loop, indicating the presence of more slit-shaped pores, consistent with the SEM results (Fig. 2). The pore size distribution curves confirm the presence of abundant 5–25 μm pores in all three aerogels. The specific surface areas of CCBA-1, CCBA-2, and CCBA-3 are 41.28, 48.36, and 46.57 m^2^/g, respectively. The superior porosity and large surface area of CCBA provide diffusion channels and abundant active sites for target metal ions, making it highly effective for practical adsorption processes.

### Static adsorption properties

#### Effect of pH and temperature

**Figure 4:**
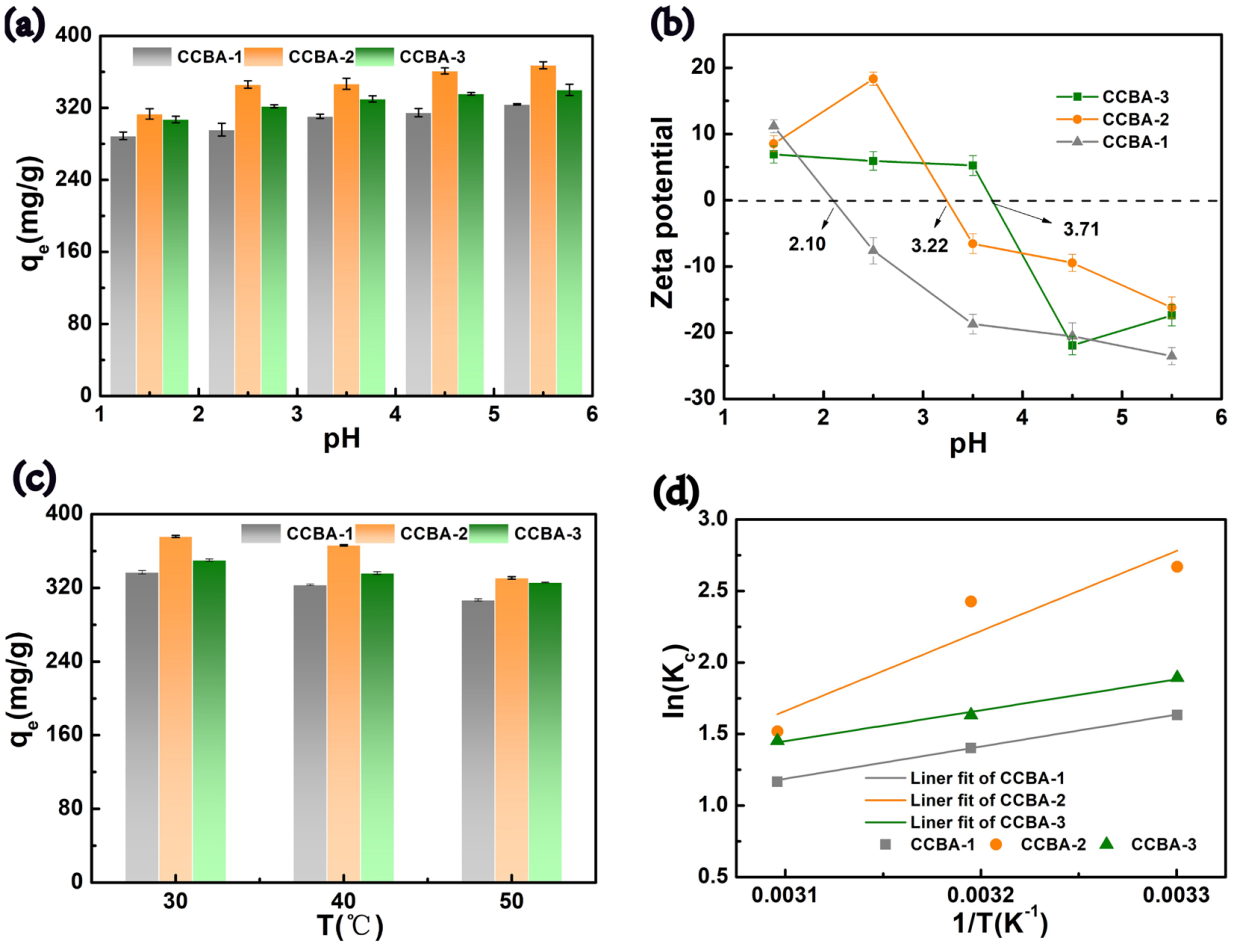
(**a**) Effect of pH to the adsorption capacity to Cu(II), (**b**) zeta potential on the CCBA (C_0_ = 400 mg/L, t = 240 min, m = 20 mg, V = 20 mL, T: room temperature), (**c**) effect of temperature on the Cu(II) adsorption capacity (C_0_ = 400 mg/L, t = 240 min, m = 20 mg, V = 20 mL, pH = 5.5), (**d**) adsorption thermodynamics (lnK_c_ versus 1/T) for Cu(II) ions on CCBA.

The pH of an aqueous solution has a significant effect on the removal of Cu(II) and can reflect the form in which the metal species exists. This affects the surface charge of CCBA and influences its chelation/electrostatic performance. Cu(II) ions are precipitated as Cu (OH)_3_^−^, Cu (OH)^+^, and Cu (OH)_2_^0^ as the pH exceeds 6^1,17^. Therefore, the experiments were carried out in a reasonable pH range of 1.5–5.5. From Fig. 4a, the adsorption capacity of Cu(II) by CCBA increased with the increasing solution pH, as the highest q_e_ of three aerogels all obtained at pH = 5.5, which may be related to the change of the surface functional group charge of the adsorbent^11^. Hence, to elucidate the effect of pH on the adsorption process, the point of zero charge (pH_pzc_) of CCBA was measured. According to Fig. 4b, the pH_pzc_ values were found to be 2.10, 3.22 and 3.71 for CCBA-1, CCBA-2 and CCBA-3, respectively. When pH < pH_pzc_, the surface of the CCBA is positively charged, resulting in electrostatic repulsion between CCBA and Cu(II) ions. Excess H^+^ in solution can also compete with Cu(II) for active sites, leading to poor adsorption. However, the adsorption amount still exceeded 72–80% as pH descended to 1.5, suggesting that the as-prepared aerogels have superior acid-resistance. When pH > pH_pzc_, the surface of CCBA is negatively charged, enhancing its complexation and electrostatic attraction ability with positive Cu(II) ions and increasing the adsorption amount. This indicates that both functional group chemical chelation and electrostatic interaction play a role in the capture of metal ions by CCBA. Therefore, the optimal pH value of 5.5 was selected for the subsequent adsorption experiments.

Furthermore, the influence of different temperatures on the adsorption process was investigated under the condition of optimal pH value of 5.5. As shown in Fig. 4c–d and Table 1, the copper adsorption capacity of CCBA-1, CCBA-2, CCBA-3 decreased from 336.96 mg/g, 375.85 mg/g, 350.25 mg/g to 307.04 mg/g, 330.87 mg/g, 325.92 mg/g, respectively, as the reaction temperature increased from 30 to 50 °C. This indicates that room temperature (30 °C) is more conducive to the adsorption process than higher temperatures. Furthermore, the △G_0_ value decreased from − 16.69 to − 42.61 kJ/mol as the temperature increased from 303 to 323 K. This suggests that the process of CCBA capturing Cu(II) ions is spontaneous^18^.

**Table 1:** Thermodynamic fitting data for the capturing of Cu(II) ions on CCBA.

T(K)	303	313	323
△G^0^ (kJ/mol)	− 16.69	− 17.24	− 42.61
Adsorbent	CCBA-1	CCBA-2	CCBA-3
△S^0^ (J/mol/K)	− 43.78	− 130.36	− 48.04
△H^0^ (kJ/mol)	− 18.01	− 46.51	− 18.68

#### Effect of contact time and initial concentration

**Figure 5:**
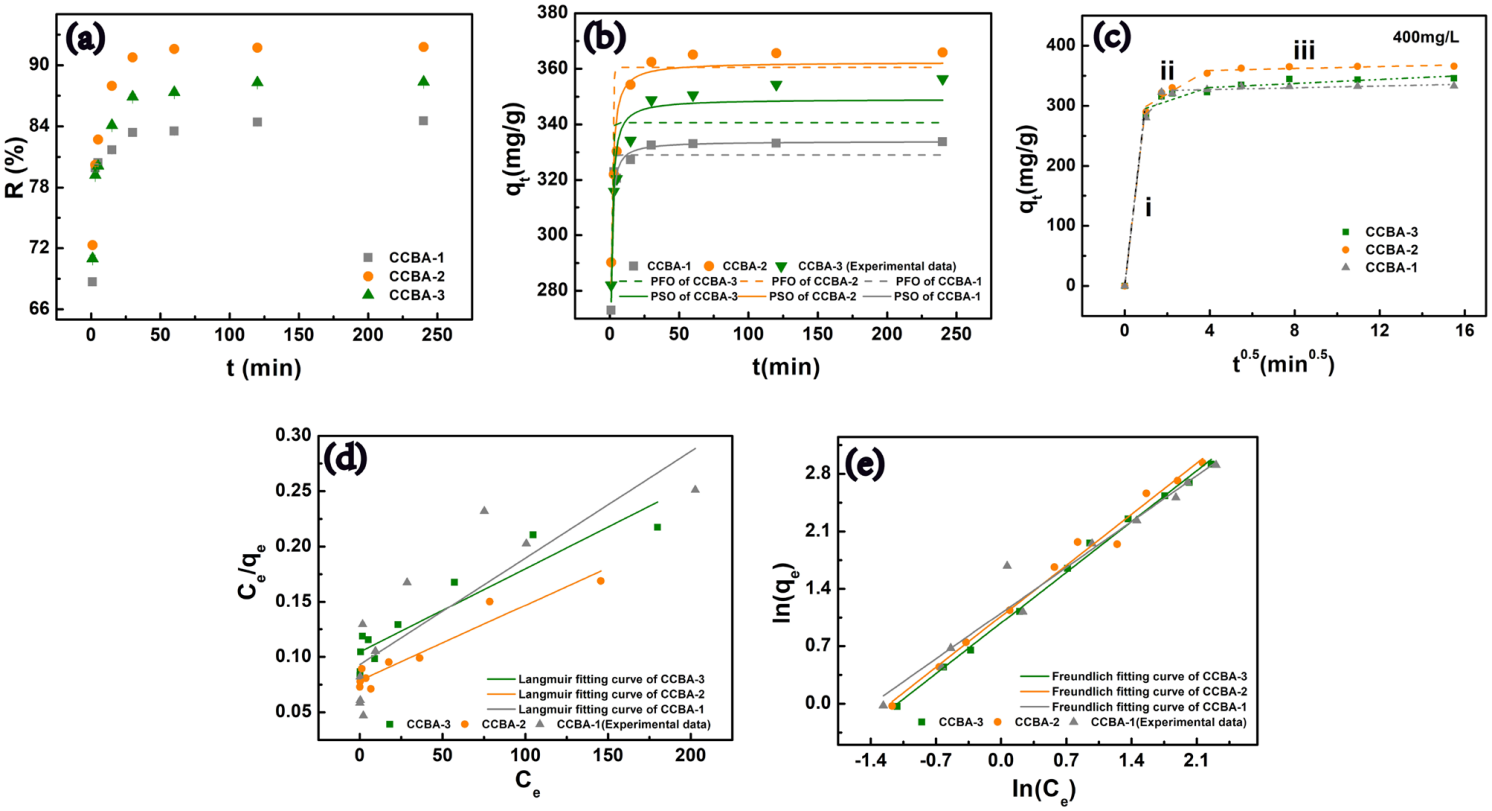
(**a**) Removing rate of Cu (II) in different contact time (C_0_ = 400 mg/L, t = 1–240 min, m = 20 mg, V = 20 mL, pH = 5.5, T = 303 K), (**b**) PFO and PSO model, (**c**) fitting results of intraparticle diffusion model, Langmuir (**d**) and Freundlich (**e**) adsorption isotherms model.

In addition, a rapid response to pollutants is essential for the application of adsorbents. Hence, we investigated the impact of contact time ranging from 1 to 240 min for the removal of Cu(II) ions under an optimum pH value of 5.5 and a temperature of 303 K. From Fig. 5a, it can be observed that the copper ions were rapidly captured by CCBA within the first 30 min. Subsequently, the adsorption process tends to stabilize, and the adsorption equilibrium is reached at 240 min. This phenomenon can be explained by the presence of numerous adsorption sites on the surface of the adsorbent initially, leading to the rapid capture of the adsorbate. As the effective binding sites become consumed, the adsorption process gradually reaches a balance^52^. In general, the complexation adsorption mechanism is slower compared to the ion exchange and hydrogen bond reaction mechanisms for adsorbing the target ion^53,54^. In the final equilibrium stage, the removal rates of copper ions by CCBA-1, CCBA-2, and CCBA-3 were 84.6%, 91.8%, and 88.4%, respectively.

According to Fig. 5b and Table 2, the PSO (0.905 ≤ R^2^ ≤ 0.973) fits the kinetic data better than PFO (0.589 ≤ R^2^ ≤ 0.919). Moreover, the fitting value of q_f2_ is much closer to the experimental value q_e(exp)_, revealing that the adsorption of Cu(II) ions by CCBA is controlled by chemisorption. Chemical interactions of chelation and electrostatic attraction are considered the main factors for Cu(II) capturing on the aerogel surfaces^2^. Figure 5c illustrates the fitting results of the intraparticle diffusion model. The three slope lines observed throughout the entire contact time indicate that different stages constitute the rate-limiting steps in the Cu(II) adsorption process. Step i demonstrates the rapid movement of Cu(II) ions from the solution to the surface of the aerogels, with the steep slope indicating the fastest adsorption process. Step ii involves the diffusion process of Cu(II) ions from the adsorbent surface to the inner macropores. Step iii corresponds to the micropore diffusion or equilibrium stage, which exhibits a slower removal rate due to higher mass transfer resistance until reaching the equilibrium point^26,55^. These findings suggest that the adsorption of Cu(II) ions on CCBA involves complex mechanisms, including chemisorption and intraparticle-diffusion processes.

**Table 2:** Adsorption parameters of Cu(II) on CCBA for the PFO and PSO.

C_0_ (mg/L)	Adsorent	q_e(exp)_ (mg/g)	PFO			PSO		
			k_1_ (1/min)	q_f1_ (mg/g)	R^2^	k_2_ (g/mg/min)	q_f2_ (mg/g)	R^2^
400	CCBA-1	333.26	1.9074	329.05	0.919	0.01669	333.93	0.958
	CCBA-2	365.89	1.5032	360.51	0.841	0.00976	362.41	0.920
	CCBA-3	345.92	1.7221	340.65	0.661	0.01081	349.15	0.905

As shown in Supplementary Fig. S4, with an increase in the initial concentration of Cu(II) ions, the adsorption capacity of CCBA also increases, but it does not reach adsorption equilibrium even at 1000 mg/L. This is due to the driving force that helps overcome the mass transfer resistance from the bulk liquid phase to the solid material. As a result, CCBA exhibits a greater potential for trapping Cu(II) ions^56^. According to Fig. 5d–e and Table 3, the Freundlich model (R^2^_CCBA-1_ = 0.957, R^2^_CCBA-2_ = 0.986, R^2^_CCBA-3_ = 0.994) fits the adsorption process better than the Langmuir model (R^2^_CCBA-1_ = 0.692, R^2^_CCBA-2_ = 0.921, R^2^_CCBA-3_ = 0.859), indicating that the Cu(II) ions adsorption by aerogels is a complex multilayer process. This is consistent with previous studies where Cu(II) adsorption by –NH_2_ and –OH functionalized aerogels followed the Freundlich isotherm^18^. The value of n in the Freundlich model is greater than 1, suggesting that the sorption process proceeds easily and co-adsorption exists^41,57^. The value of q_m_ in Langmuir isotherms for CCBA-2 reaches 1468 mg/g, which is well above the experimental values (861.58 mg/g), indicating that CCBA-2 has a high potential to adsorb Cu(II). Therefore, CCBA-2 is selected for subsequent experiments.

**Table 3:** Adsorption parameters of Cu(II) on CCBA for the Langmuir and Freundlich isotherms model.

Adsorent	q_e_ (mg/g)	Langmuir			Freundlich		
		k_L_ (L/mg)	q_m_ (mg/g)	R^2^	k_F_ (mg/g)	n	R^2^
CCBA-1	806.76	0.010354	1037.34	0.6916	3.0148	1.254	0.9573
CCBA-2	861.58	0.008657	1468.43	0.9206	2.9001	1.126	0.9864
CCBA-3	827.06	0.007225	1324.50	0.8497	2.6740	1.132	0.9957

Table 4 lists various adsorbents for removing Cu(II) in water matrices. A comparative study shows that the q_e_ of CCBA is significantly higher than that of other materials such as mesoporous silica^19^ and composite material^7^, graphene oxide/montmorillonite composite aerogel^3^, MnFe_2_O_4_-cellulose magnetic composite aerogel^22^, MCC-PDA-PEI/CS-PDA-PEI hydrogel beads^2^, naste reed-based aerogel^18^, lignocellulose-based composite hydrogel^58^, nanocellulose-based polyethylenimine aerogels^1^, cellulose nanofiber/chitosan/montmorillonite aerogel^49^. In contrast to some of the petroleum precursors used in the above compounds (graphene oxide, silicon), CS and BT are natural polymers, and CP can be recycled for waste treatment, all three are easy to obtain, environmentally friendly and non-toxic. Combined with the simple synthesis route of CCBA aerogels and high adsorption yield, this adsorbent may be a potential material for trapping Cu(II) in effluent.

**Table 4:** Comparison of Cu(II) adsorption on CCBA and similiar adsorbents.

Absorbent	q_max_ (mg/g)	References
Graphene oxide/montmorillonite composite aerogel	101.83	^3^
MCC-PDA-PEI/CS-PDA-PEI hydrogel beads	434.8	^2^
Amino-modified carboxymethyl chitosan aerogel	175.56	^9^
Nanocellulose-based polyethylenimine aerogels	485.44	^1^
Lignocellulose-based composite hydrogel	541.00	^58^
Cellulose nanofiber/chitosan/montmorillonite aerogel	181.92	^49^
Graphene oxide/montmorillonite composite aerogel	101.83	^3^
Carboxymethyl cellulose/PEI	294.79	^8^
CMC/PAM composite hydrogel	227.3	^51^
MnFe_2_O_4_-Cellulose magnetic composite aerogel	63.3	^22^
Chitosan-montmorillonite composite aerogel	86.95	^17^
Waste reed-based aerogel	260.41	^18^
Citrus fruit residues based active carbon	69.69	^59^
Konjac glucomannan/chitosan aerogels	184	^39^
Untreated waste office paper-chitosan based aerogel	156.3	^37^
Chitosan-orange peel-based hydrogel	116.64	^41^
Hydrogels composite material	171.3	^7^
HMBA immobilized mesoporous silica monoliths	182.39	^15^
Mesoporous silica based meso-adsorbent	175.75	^19^
CCBA-1	806.76	This study
CCBA-2	861.58	This study
CCBA-3	827.06	This study

#### Effect of selectivity and coexisting ions

**Figure 6:**
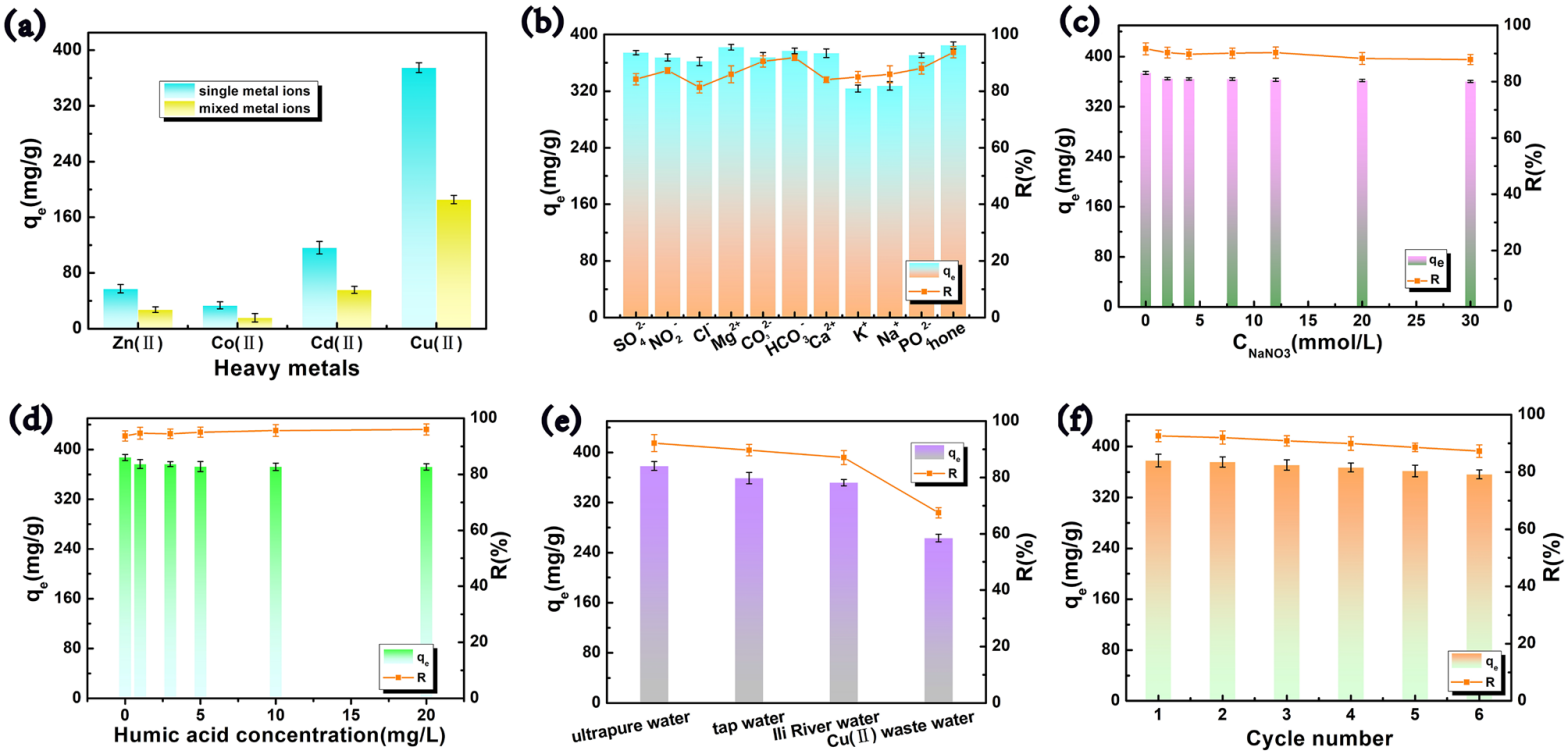
(**a**) Selectivity adsorption in single and mixed system (t = 240 min, pH = 5.5, m = 20 mg, V = 20 mL, T = 303 K), coexisting ions (**b**), ion strength (**c**), humic acid concentration (**d**), different water matrices (**e**), and cycle number (**f**) on Cu(II) removal (C_0_ = 400 mg/L, t = 240 min, pH = 5.5, m = 20 mg, V = 20 mL, T = 303 K).

Adsorption selectivity is a crucial metric for evaluating the effectiveness of adsorbents in separating target pollutants. As can be seen in Fig. 6a, in a single metal ion system with an initial concentration of 400 mg/L for each heavy metal, the adsorption of Cu(II) by CCBA-2 is the highest (374.92 mg/g). In a mixed metal ion system with composite pollution (C_Cu(II)_: 395.2 mg/L, C_Zu(II)_: 388.4 mg/L, C_Cd(II)_: 387.6 mg/L, C_Co(II)_: 375.5 mg/L), the adsorption amount of 185.33 mg/g for Cu(II) ions by CCBA-2 is still significantly higher than that of other metals in the same system. Moreover, the selectivity coefficient (K_Cu/M_) can quantitatively analyze the selectivity property of CCBA-2 for Cu(II). Generally speaking, a larger K_d_ value indicates a higher adsorption capacity of the adsorbent, while smaller K_d_ values mean that most of the metal ions are not adsorbed and remain in the solution^43^. From Table 5, the K_d_ value follows the sequence: Cu^2+^(1138.5) > Cd^2+^(76.4) > Zn^2+^(39.3) > Co^2+^(19.3), indicating that CCBA-2 has a much stronger affinity for Cu(II). The corresponding calculation for α^Cu^ (all > 1) demonstrates that CCBA-2 has superior selective adsorption capacity for Cu(II) ions. This phenomenon may be attributed to that the higher electronegative value of Cu (1.9) compared to Co (1.88), Zn (1.65), and Cd (1.69), which makes it more likely to attract electrons in the compound. Another reason could be the radius of their hydrated ions, with R_[Cu(H2O)4]_^2+^(4.19) < R_[Co(H2O)6]_^2+^(4.23) < R_[Cd(H2O)4]_^2+^(4.26) < R_[Zn(H2O)6]_^2+^(4.30). The smaller radius favors the binding of copper ions to the active site in CCBA-2^3,60^.

**Table 5:** Selective adsorption parameter of CCBA-2.

Metal ions	C_b_ (mg/L)	C_f_ (mg/L)	K_d_^M^ (mL/g)	α^Cu^ (K_d_^Cu^/K_d_^M^)
Cu(II)	395.2	184.8	1138.5	1
Zn (II)	388.4	373.7	39.3	29.0
Cd (II)	387.6	360.1	76.4	14.9
Co (II)	375.5	368.4	19.3	59.1

In addition, the composition of actual water matrices or industrial wastewater may contain a variety of common anions/cations and one or more metal cations, such as HCO_3_^−^, CO_3_^2−^, PO_4_^2−^, NO_2_^−^, SO_4_^2−^, Cl^−^, K^+^, Ca^2+^, Mg^2+^ and Na^+^. It's crucial to understand the effect of these coexisting ions on the adsorption process. In this study, we maintained the concentration of coexisting cations and anions at 20 mmol/L, as shown in Fig. 6b. For coexisting anions, their effect on copper capture by CCBA-2 is minimal and can be essentially ignored. However, in the presence of four cations, especially K^+^ and Na^+^, they do have a certain impact on the removal process of Cu(II) ions by CCBA-2. This is mainly because they compete with Cu(II) ions for adsorption sites to some extent^9^. Despite this competition, CCBA-2 still exhibits a high adsorption capacity of 323.79 mg/g, and the removal rate of Cu(II) by the aerogel remains above 80%. This characteristic is beneficial for the practical use of CCBA-2 in complex water pollution matrices.

Sodium, commonly found in water matrices, can influence the adsorption process by competing for binding sites through electrostatic effects or by forming complexes with target metal ions^61^. Figure 6c shows the effect of ionic strength on Cu(II) adsorption. As the concentration of NaNO_3_ increases, the removal rate of Cu(II) slightly decreases. This suggests that the adsorption process is generally independent of the liquid's ionic strength and primarily depends on the chemical chelation between the adsorbent and the adsorbate. These results confirm that the synthesized aerogel has excellent adaptability in various complex water matrices.

#### Effect of organic matter and complex water matric

Natural water/wastewater matrices contain various organic compounds that may cause synergistic or competitive adsorption with Cu(II). Humic acid (HA), a common natural organic compound, was chosen for this study. As shown in Fig. 6d, the adsorption of Cu(II) slightly decreases as the concentration of HA increases from 0 to 20 mg/L. HA, derived from plant residues, has numerous functional groups and exhibits a negatively charged surface when the pH is greater than 2^62^. Given that the pH_pzc_ of CCBA-2 is 3.22 (Fig. 4b), the negatively charged CCBA-2 exerts a repulsive force on HA in neutral aqueous media, thereby reducing HA's inhibitory effect. The complex surface heterogeneity may also reduce the adsorption capacity.

The adsorption response of adsorbents to pollutants in real water is also an aspect that needs to be investigated to expand its practical application. Therefore, three water matrices were tested: tap water, Ili River water, and simulated actual copper-containing wastewater, with the specific parameters listed in Supplementary Table S2. According to Fig. 6e, the adsorption capacity of CCBA-2 for Cu(II) ions in tap water and Ili River water is 359 mg/g and 351.9 mg/g, respectively, which is similar to the value in ultrapure water (q_e, Up_ = 378.34 mg/g). However, the adsorption capacity in simulated actual wastewater is 263.4 mg/g, indicating that the complex ion composition in the liquid can compete with Cu(II) for effective adsorption sites. Despite this, CCBA-2 still achieves a copper removal rate of close to 60% in simulated wastewater, demonstrating its potential for application in actual water matrices. Further research is needed to optimize the modification of the adsorbent and improve its adsorption and recovery performance for heavy metal ions in complex water matrices.

#### Elution and regeneration

To assess the economic applicability of adsorbents, cycle sustainability, renewability, and reuse are key indicators^63^. Low concentrations of HNO_3_ (1 M) can elute and regenerate CCBA-2 saturated with Cu(II), retaining its initial function. In this study, 20 mg of the composite adsorbent was immersed in 20 mL of 400 mg/L Cu(II) solution and stirred for 240 min at pH 5.5 to reach adsorption equilibrium. The elution regeneration study was then conducted with a 1 M HNO_3_ solution in 20 mL solution. As shown in Fig. 6f, across five adsorption–desorption cycle experiments, the desorption efficiency of Cu(II) ions by 1 M HNO_3_ was 99.4%, 98.15%, 97.12%, 95.68%, and 94.25%, respectively. The removal efficiency of copper by the aerogel was reduced by only 5.3%, and the adsorption capacity decreased from 378 to 356.2 mg/g. This slight decrease may be due to a small number of Cu(II) ions being adsorbed on CCBA-2 by ion exchange, while the majority are captured by chemical chelation. The reusability studies show that CCBA-2 can be recycled multiple times in the polymer matrix reinforced by citrus peel and bentonite. Its internal pores are not prone to degradation and collapse, ensuring its nearly unchanged adsorption affinity for Cu(II) ions across multiple cycle experiments. Therefore, CCBA-2 is a green, sustainable, and cost-effective Cu(II) ion collector for water pollution remediation.

### Environmental applications

**Figure 7:**
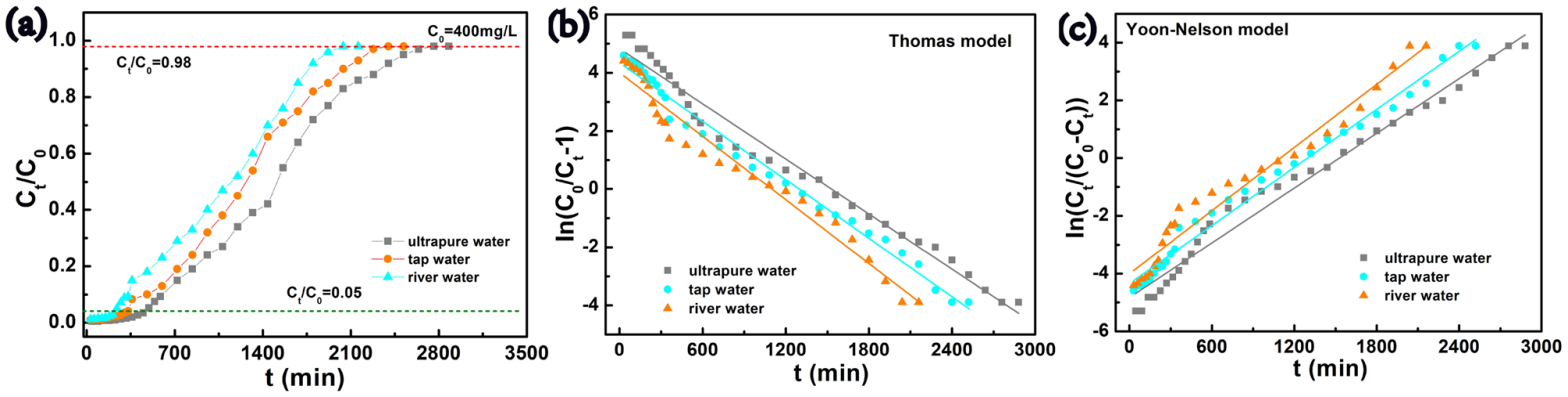
(**a**) The breakthrough curve of Cu(II) on CCBA-2, (**b**,**c**) the Thomas and Yoon-Nelson model fitting curves of the original breakthrough data obtained from the fixed-bed column.

Given its superior adsorption and regeneration properties in complex water mediums, CCBA-2 can be considered as an adsorbent for compact filtration devices in practical applications. The fixed-bed column adsorption is an effective implementation method, with the simple experimental setup and relevant column parameters presented in Supplementary Fig. S5 and Supplementary Table S3. As shown in Fig. 7a, with an influent concentration of 400 mg/L, the t_b_ and t_e_ of ultrapure water (Up) always appeared the latest, while those of Ili River water appeared the earliest. This is positively correlated with the complexity of water quality. The more complex the water matrix, the more mixed ions compete with Cu(II) for adsorption sites, suggesting that the adsorption process could be negatively affected by the increase in water substrates complexity^61,64^. The adsorption process and the breakthrough curve were fitted by the Thomas and Nelson models with the influent concentration of 400 mg/L for Cu(II) in a fixed-bed column. The results displayed in Table 6 and Fig. 7b,c. The parameter of adsorption capacity in the dynamic system (q_Th_) followed the trend of q_Th, Up_ > q_Th, tap_ > q_Th, river_, which was negatively correlated with the complexity of water quality. However, the removal capacity of the fixed-bed column based on tap water and river water was only 1.2% and 1.3% less than the column based on ultrapure water. This demonstrates that CCBA-2 could be applied in the continuous treatment of complex water matrices with metal ions pollution. The adsorption amount of q_e_, ranging from 277.94 to 377.40 mg/g in the dynamic fixed-column test, was not as desirable, mainly due to the high inlet concentration and the short contact time between CCBA-2 and the influent^65,66^. In practical applications, it is suggested to prolong the contact time between the treatment solution and the adsorbent, or use a series column adsorption experimental device to improve the removal rate. Although the fitting value of R^2^ in both models was greater than 0.97, the fitted values τ_Yn_ (C_t_/C_0_ = 0.50) in the Yoon-Nelson model were basically equal to the experimental values τ_e_. This demonstrates that the breakthrough curve and adsorption process of CCBA-2 for Cu(II) adsorption on a fixed-bed column fit well with the Yoon-Nelson model.

**Table 6:** Thomas and Yoon-Nelson model parameters obtained from breakthrough curves for different matrices in the fixed-bed column experiment.

Water matrix	C_0_ (mg/L)	R (%)	q_e_ (mg/g)	Thomas model				Yoon-Nelson model		
				K_Th_ (mL/(min·mg))	q_Th_ (mg/g)	R^2^	K_Yn_ (min^−1^)	τ_Yn_ (min)	τ_e_ (min)	R^2^
Up	400	52.4	377.40	0.0078	415.48	0.98	0.0032	1509	1500	0.98
Tap water	400	51.2	320.71	0.0083	350.71	0.99	0.0034	1276	1250	0.99
River water	400	51.1	277.94	0.0090	297.04	0.97	0.0037	1084	1100	0.97

From Supplementary Fig. S6, it's observed that for the fixed-bed column with tap water influent first adjusted to 10 mg/L (C_Cu(II)_), the concentration of effluent remained below the limit values of the Farmland Irrigation Water Quality Standard (1 mg/L, GB5084-2021) even when the volume of effluent reached 20 L. The comprehensive utilization and ecological risk of this low concentration effluent were explored through pot experiments, please see the support information (SI) and Supplementary Fig. S7 for details. Furthermore, considering that CP, CS, and BT are all green and non-polluting base materials, CCBA-2 could be dispersed on agricultural or forest land after its repeated use and complete desorption and purification. Assuming that the disposal phase does not cause secondary pollution to the environment, it could even serve as a sustainable fertilizer delivery system^67^.

### Adsorption mechanisms of Cu(II)

**Figure 8:**
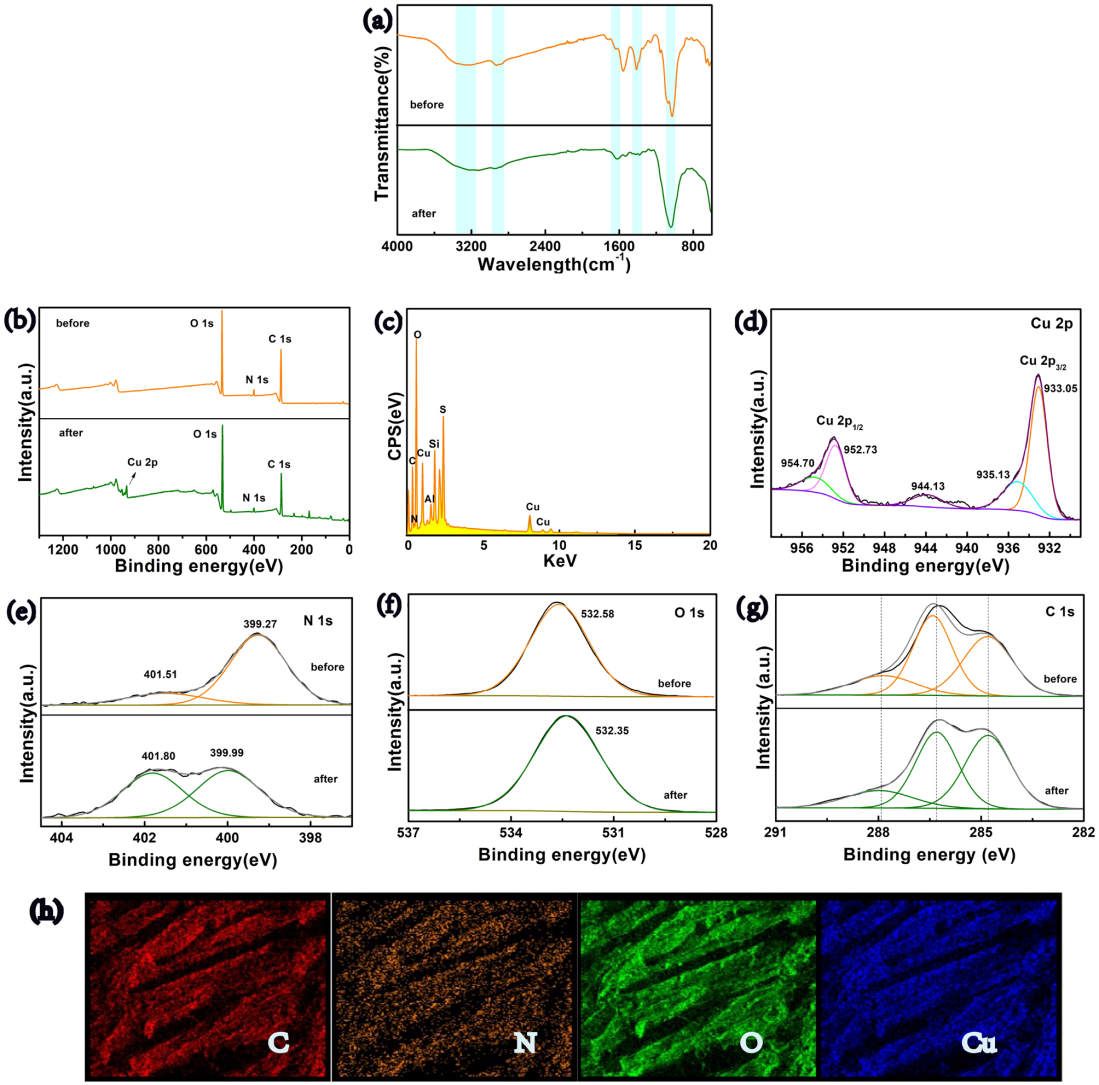
(**a**) FTIR spectra of CCBA-2 before and after Cu(II)-loading, (**b**) Full XPS spectrum of CCBA-2 before and after Cu(II) adsorption, (**c**) EDS analysis of CCBA-2 after Cu(II) adsorption, high-resolution spectra of Cu 2p (**d**), N1s (**e**), O1s (**f**) and C1s (**g**) of CCBA-2 before and after Cu(II) adsorption, (**h**) elements images of EDX mapping for C, N, O, and Cu.

To further clarify the adsorption mechanism, FTIR, EDS, and XPS were conducted. According to Fig. 8a, the peak at 3225 cm^−1^, associated with the stretching vibration of –COOH/–OH, shifted to 3193 cm^−1^ after Cu-loading. This indicates an increase in electron density due to the coordination of these functional groups with copper ions. The stretching vibration of –C–H at 2927 cm^−1^ also moved to 2947 cm^−1^, and its peak intensity weakened. Moreover, characteristic peaks of amide I and amide II at 1633 cm^−1^ and 1555 cm^−1^ shift to 1626 cm^−1^ and 1527 cm^−1^, respectively, and their peak intensities weakened after Cu(II) loading. These observations provide evidence of strong chemical chelation between Cu(II) ions and functional groups such as –NH_2_ or –OH^9,18^.

As depicted in the full XPS spectrum of CCBA-2 after Cu(II) sorption (Fig. 8b), the presence of the Cu 2p peak confirms the successful immobilization of Cu(II) on the aerogel^3^. This is further supported by the Cu(II) characteristic peak in the EDS spectrum (Fig. 8c). In Fig. 8d, two main peaks are observed in the Cu 2p adsorption peak: one at 933.05 eV (Cu 2p_3/2_), attributed to the complexes of (NH_2_)_2_Cu^2+^ or NH_2_Cu^2+^, and the other at 952.73 eV (Cu 2p_1/2_), assigned to pure Cu physisorptions^8^. The N 1 s high-resolution spectrum (Fig. 8e) shows two peaks at 399.27 eV and 401.51 eV, indicating the presence of –NH_2_ and –NH groups, respectively. Both bands slightly shifted to higher binding energy. Likewise, the O 1 s (Fig. 8f) also shifts to a lower binding energy after adsorption with an offset of about 0.23 eV. These shifts suggest the formation of coordination bonds like O → Cu and N → Cu due to the presence of lone pair electrons from O and N^1,2^, indicating that both coordination and oxidation reactions occur in the adsorption process. However, the displacement changes of the C 1 s (Fig. 8g) before and after Cu(II) adsorption are small. EDS spectroscopy (Fig. 8h) further supports these findings, showing that Cu elements are uniformly distributed on the surface of CCBA-2 without loss. This suggests that Cu(II) ions are successfully anchored on the active sites (–NH_2_ and –OH) in the aerogel.

**Figure 9:**
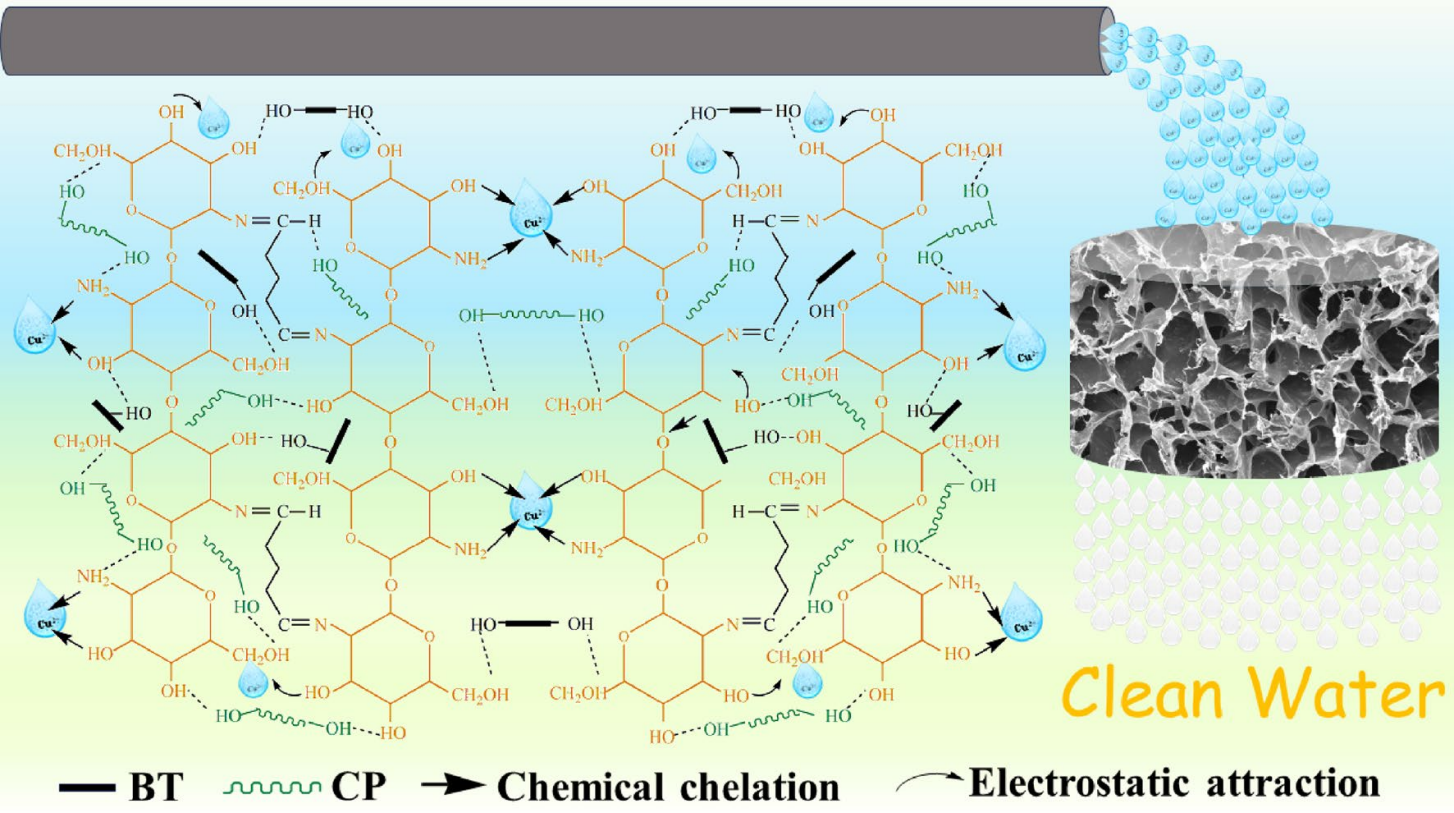
Proposed mechanism of CCBA to Cu(II) ions.

Based on the analysis, the adsorption mechanism of CCBA-2 to Cu(II) is proposed in Fig. 9. The majority of Cu(II) is fixed by CCBA-2 primarily through chemical chelation. Additionally, the presence of BT in CCBA-2 results in a negatively charged surface at pH = 5.5, which can attract positively charged Cu(II) ions electrostatically. Therefore, the capture of metal cations by CCBA-2 is mainly dominated by electrostatic attraction and chemical chelation.

In conclusion, the efficient trapping ability of CCBA-2 for Cu(II) ions is attributed to the unique physicochemical structure of the material. The regular and dense 3D interpenetrating porous structure provides an efficient transport channel for the rapid adsorption of copper ions. The addition of CP and BT significantly improves the mechanical properties and water stability of the entire aerogel system. CS, as a functional group supplier, provides abundant -NH_2_ and -OH groups that can form a bidentate or tetradentate chelate ligand with copper ions^1^. All these factors contribute to the effective capture of Cu(II).

## Materials and methods

### Materials and characterization

All reagents and specific characterization methods used in this experiment are detailed in the supporting information (SI).

### Synthesis of CCBA

First, dried the collected citrus peels at moderate temperature (30 °C), then crushed them with a grinder, sieved through 100 mesh, and placed them in a dryer for further use. Subsequently, prepare a chitosan solution (CS, 2 wt%) by dissolving 10.0 g CS in 490 mL of acetic acid solution (1%, v/v) with continuous stirring until the CS powder dissolves. Meanwhile, BT/CP with mass ratios of 2:1, 1:1 and 1:2 were placed in 30 mL of ultrapure water and thoroughly dispersed, respectively. Then, mix them all with an equivalent volume of chitosan solution (CS, 2 wt%), polyvinyl alcohol (PVA, 3 wt%) and glutaraldehyde (GA, 2.5%, v/v), separately, and mechanically stir the mixture until the solution becomes almost a colloidal gel. Pour the sample into a round sample box with a lid (D = 1 cm, H = 1.2 cm), and immediately place it in the cold trap of a pre-cooled freeze dryer for freezing (− 82 °C). About half an hour later, take them out and put them on the sample table to freeze and dry. At that time, the vacuum degree of the freeze dryer is about 1.3. The drained samples should be soaked with anhydrous ethanol to remove the incomplete reaction of organic substances in the material, and then cleaned with ultrapure water several times, and lyophilized under the same conditions to obtain CCBA. The aerogels obtained by the three mass ratios are named as CCBA-1 (m_BT_:m_CP_ = 2:1), CCBA-2 (m_BT_:m_CP_ = 1:1), and CCBA-3 (m_BT_:m_CP_ = 1:2), respectively. For the sake of comparison, the pure CS aerogel (CSA) and CS/CP aerogel (CSPA) were also fabricated according to the above-mentioned methods without added CP/BT or BT.

### Static adsorption experiments

Static adsorption experiments were performed in a 100 mL conical flask, and the adsorption process was carried out by adding 0.02 g of aerogel to 20 mL of Cu(II) solution, with HNO_3_ (0.1 M) or NaOH (0.1 M) used to adjust the pH value of the solution from 1.5 to 5.5. The concentration of Cu(II) ions in liquid supernatant was measured by the BCO-Spectrophotometry method at 602 nm (UV-1200, MAPADA, China; Calibration curves are placed in SI, Supplementary Fig. S8). The adsorption capacity (q_e_, mg/g) and removal capacity (R, %) of the aerogel were calculated according to the following equations:

1 $${q}_{e} (mg/g) = \frac{{c}_{0}-{c}_{e}}{m} \times V$$

2 $$R (\%) = \frac{{C}_{0}-{C}_{e}}{{C}_{0}} \times 100$$

where C_0_ and C_e_ (mg/L) are the initial and equilibrium concentrations of metal ions in the solution, respectively. V (L) is the volume of the solution and m (g) is the mass of CCBA.

*Adsorption thermodynamics* experiment were measured under 303, 313, 323 K with the initial concentrations of 400 mg/L for 240 min. Cu(II) ions at beginning and equilibrium stages were used to calculate the thermodynamic constants according to the following formula:

3 $$\ln K_{c} = \frac{{\Delta S^{0} }}{R} - \frac{{\Delta H^{0} }}{RT}$$

4 $$K_{c} = \frac{{C_{0} - C_{e} }}{{C_{e} }} \times \frac{V}{m}$$

5 $$\Delta G^{0} = \Delta H^{0} \, - \,T\Delta S^{0}$$

where the values of *ΔH*^*0*^ (kJ/mol) and *ΔS*^*0*^ (J/mol/K) are the changes of enthalpy and entropy, respectively, which can be obtained from the slope and intercept of lnK_d_ versus 1/T plot. T is the temperature in K, and R is the universal gas constant (8.314 J/mol). From the initial and equilibrium concentrations of Cu(II) ions, the distribution coefficient (K_c_) can be calculated. V(L) is the volume of the Cu(II) solution used, and m(g) is the mass of the adsorbent. *ΔG*^*0*^ (kJ/mol) is the change of Gibbs free energy.

*Adsorption kinetics* experiment were performed with initial concentrations of 400 mg/L for 1–240 min. To better understand the adsorption process, three kinetic models including the pseudo-first-order (PFO, Eq. 6), the pseudo-second-order (PSO, Eq. 7) and the intra-particle diffusion (IPD, Eq. 8) models were applied to fit the experimental data and analyze the adsorption mechanism.

6 $${q}_{t}={q}_{f}\left(1-exp\left(-{k}_{1}t\right)\right)$$

7 $${q}_{t}=\frac{{k}_{2}{q}_{f}^{2}t}{1+{k}_{2}{q}_{f}t}$$

8 $${q}_{t}={k}_{p}{t}^{0.5}+C$$

where k_1_ (min^−1^), k_2_ (g/mg/min) and k_p_ (mg/(g·min^0.5^)) are the adsorption rate constant for the PFO, PSO and IPD fitting model, respectively. C is the intercept for the intraparticle diffusion model, q_f_ (mg/g) is the fitted adsorption value at equilibrium, and q_t_ (mg/g) is the experimental value at a set time t (min), respectively.

*Adsorption isotherm* test was performed at concentration within 1–1000 mg/L for 240 min at 303 K. To delve deeper into this adsorption behavior, the experimental data were fitted by Langmuir (Eq. 9) and Freundlich (Eq. 10) isotherm models:

9 $$\frac{{C}_{e}}{{q}_{e}}=\frac{1}{{q}_{m}}{c}_{e}+\frac{1}{{q}_{m}{k}_{L}}$$

10 $${Inq}_{e}={Ink}_{F}+\frac{1}{n}{Inc}_{e}$$

where q_m_ (mg/g) is the maximum adsorption capacity, C_e_ (mg/L) is the equilibrium concentration of metal ions, K_L_ (L/mg) and K_F_ ((mg/g)/(mg/L)/n) are constants related to adsorption, and n is the Freundlich constant related to the adsorption capacity. Usually 2 ≤ n ≤ 10 leads to absorption, 1 ≤ n < 2 represents moderate absorption, and n < 1 indicates difficult absorption^57^.

*Selectivity coefficient (K*_*Cu*_*/M)* is used to evaluate the selective removal ability of the adsorbent for target metal ions in multi-component solutions, which can express by the following equations:

11 $${K}_{d}=\frac{({C}_{b}-{C}_{f})}{{C}_{f}}\times \frac{V}{m}$$

12 $${\alpha }_{M}^{{C}_{u}}=\frac{{K}_{d}^{{C}_{u}}}{{K}_{d}^{M}}$$

where C_b_ (mg/L) and C_f_ (mg/L) are the initial and final concentrations of metal ions, m (g) is the weight of the CCBA, V (mL) is the volume of the aqueous solution, while ${K}_{d}^{M}$ and ${K}_{d}^{Cu}$ are the interfering metal ions and distribution coefficient of Cu(II) ions, respectively.

### Dynamic adsorption experiments

In the dynamic adsorption experiments, a series of fixed-bed column adsorption experiments were carried out. Two different water matrices (tap water and Ili River (81° 18′ N, 43° 53′ E)) were selected as the research cases. A Cu(II) ions stock solution (1000 mg/L) was used to adjust the influent to 400 mg/L before the experiment. The effluent was collected at different time intervals (time from the first drop). To predict the fixed-bed column breakthrough curves (BTC), the Thomas model (Eq. 13) and Yoon-Nelson model (Eq. 14) were used to fit the experimental data of the dynamic column adsorption.

13 $$In\left(\frac{{C}_{0}}{{C}_{t}}-1\right)=\frac{{K}_{Th}{q}_{Th}\times m}{{Q}_{v}}-{K}_{Th}{c}_{0}t$$

14 $$In\left(\frac{{C}_{t}}{{C}_{0}-{C}_{t}}\right)=t{K}_{Yn}-{\tau }_{Yn}{K}_{Yn}$$

where C_0_ and C_t_ (mg/L) are the influent and effluent concentrations of Cu(II), respectively, K_Th_ (mL/min/mg) is Thomas kinetic constant, m (g) is the total mass of the aerogel in the column, q_v_ (mL/min) is the volumetric flow rate, t is the flow time (min), and q_Th_ (mg/g) is the absorption capacity in dynamic system. K_Yn_ (min^−1^) and τ_Yn_ (min) are the rate constant of the Yoon-Nelson model and the time for the effluent Cu(II) ions concentration to reach half of the inlet concentration (C_t_/C_0_ = 0.5), respectively.

Meanwhile, the t_b_ and t_e_ (min) were obtained by the BTC when the C_t_/C_0_ reached to 0.05 and 0.98, respectively. In addition, the total adsorption amount (q_total_, mg) of Cu(II), the adsorption capacity in equilibrium (q_e_, mg/g) and the removal efficiency (R, %) were calculated by following equations^18^:

15 $${q}_{total}=\frac{{q}_{v}}{1000}{\int }_{t=0}^{t={t}_{total}}{C}_{s}dt$$

16 $${q}_{e}=\frac{{q}_{total}}{m}$$

17 $$R=\frac{1000\times {q}_{total}}{{C}_{0}\times {q}_{v}{t}_{total}}$$

where A is the area under the BTC, t_total_ (min) is the total adsorption time, and C_sp_ (mg/L) is the difference between the values of Cu(II) in the influent and effluent (C_sp_ = C_0_-C_t_).

Meanwhile, taking tap water as the medium, the penetration curve of fixed-bed column to low concentration copper wastewater (10 mg/L) was investigated. Besides, the effluent (tap water) from above mentioned fixed-bed column test was taken as the medium of hydroponics, and mung bean was taken as the object of hydroponics to preliminarily explore the comprehensive utilization way and ecological risk of the effluent. In the blank control experiment, the tap water was without adding extra Cu(II) source, other conditions being the same. All the culture processes were performed under the same conditions, including humidity, temperature and light.

### Desorption and regeneration

Please refer to SI for detailed information.

## Conclusion

In this study, a green and sustainable CCBA aerogel was successfully developed as an effective adsorbent for the selective removal of Cu(II) ions from wastewater. The CP and BT substrates provided excellent mechanical capacity, while CS acted as a functional component of the adsorbent. The CCBA-2 exhibited a maximum adsorption capacity of 841.58 mg/g for Cu(II) ions. Meanwhile, the adsorption kinetics followed the pseudo-second-order model, and the isotherm conformed to the Freundlich model, indicating a multilayer chemisorption process. Furthermore, in a mixed metal ions system containing Zn(II), Co(II), Cd(II), and Cu(II), the CCBA aerogels demonstrated the highest selectivity coefficient for Cu(II) ions. Factors such as coexisting cations/anions, ionic strength, organic matter, and water quality had an insignificant effect on the removal of copper ions by CCBA-2. The fixed bed column adsorption experiment confirmed the efficient removal of copper ions from complex water matrices using CCBA-2. Based on experimental and characterization analysis of CCBA-2 before and after Cu(II) loading, the adsorption mechanism was identified as electrostatic attraction and chemical chelation between the functional groups (-NH_2_, -OH) of the aerogel and Cu(II) ions. CCBA-2, saturated with Cu(II) ions, could be regenerated into its initial form by elution with 1 M HNO_3_ for subsequent operations, and it exhibited stable performance over five cycles of adsorption–desorption without significant degradation. Overall, the synthesized CCBA-2 aerogel proves to be a cost-effective and sustainable adsorbent, offering a promising alternative for the efficient and environmentally friendly removal of toxic Cu(II) ions from wastewater.

### Supplementary Information

Supplementary Information 1.

Supplementary Information 2.

## Data Availability

Data will be made available on request by asking for the corresponding author.
