# Supplementary material for: Green composite aerogel based on citrus peel/chitosan/bentonite for sustainable removal Cu(II) from water matrices
Source: Sci Rep. 2023 Sep 18;13:15443. doi: 10.1038/s41598-023-42409-2 (PMC10507072; doi:10.1038/s41598-023-42409-2)
Supplement: Supplementary file 1 — Supplementary Information 1. [file 41598_2023_42409_MOESM1_ESM.pdf]

# Supporting information

**Green composite aerogel based on citrus peel/chitosan/bentonite for sustainable removal Cu (II) from water matrices**

Jing Nie[a,*], Dan Feng[a], Jiangwei Shang[a], Bate Nasen[b], Tong Jiang[a], Yumeng Liu[a], Siyi Hou[a]

[a] Key Laboratory of Pollutant Chemistry and Environmental Treatment, College of Resources and Environment, Yili Normal University, Yining 835000, China

[b] College of Chemistry and Chemical Engineering, Yili Normal University, Yining 835000, China

* E-mail address of the corresponding author: X8151329@163.com

## Experimental

### Materials and characterization

Na-bentonite (BT, $Al_2O_9Si_3$, specific surface aera:240m$^2$/g), chitosan (CS, degree of deacetylation≥95%, viscosity:100-200mpa.s), glutaraldehyde (GA, 50wt% in water) and polyvinyl alcohol (PVA, alcoholysis degree: 87-89mol%, viscosity: 80–110mpa.s) were purchased from Macklin. Citrus peel (CP) collected from daily household waste in YiLi, XinJiang. Cu $(SO_4)_2 \cdot 5H_2O$ was purchased from Sinopharm Chemical Reagents Co., Ltd. All other commonly used chemicals are analytical grade and do not require further purification. Ultra-pure water was used throughout the experiment.

CCBA was lyophilized using a lyophilizer (FD-2, Biocool, Beijing). The mechanical properties of CCBA were tested using a universal testing machine (UTM-1432, Jinjian, Hebei) with a loading speed of 2 mm/min. The extremity strain was set at 75%, and the mechanical test was performed three times for each sample. The surface ultrastructural morphology of CCBA was observed using a scanning electron microscope (SEM, ZEISS Gemini 300) with an accelerating voltage of 0.02-30 kV. Energy dispersive X-ray spectroscopy (EDS, Oxford Xplore) was used to confirm the presence of Cu(II) ions on the surface of the materials at an accelerating voltage of 30 kV. The changes in functional groups before and after Cu(II) adsorption were determined by Fourier-transform infrared (FT-IR, Thermo Scientific Nicolet iS20) spectroscopy. The resolution was set at 4 cm-1, and the KBr tablet method was used with a test wavenumber range of 600-4000 cm-1. The surface elemental composition of CCBA before and after adsorption was analyzed using an X-ray photoelectron spectrometer (XPS, Thermo Scientific K-Alpha, USA) with an excitation source of Al Kα X-ray at 1486.6 eV and 400 μm. The XPS curve fitting of elements was performed using Thermo Avantage software. The specific surface area was calculated using the

Brunauer-Emmert-Teller (BET, ASAP2460) method, and the pore size distribution was determined using the BJH method.

**Desorption and regeneration**

The desorption and regeneration experiments were conducted in 20 mL of a 400 mg/L Cu (II) ions solution at pH 5.5. A total of 20 mg of CCBA was added to the solution and the mixture was kept at 30 ºC for 240 minutes. After filtration, the Cu(II)-loaded adsorbent was dispersed in 20 mL of a 1 mol/L $HNO_3$ solution and shaken at 30 ºC for 240 minutes. The solution was then neutralized using a diluted NaOH solution. The CCBA was filtered and washed multiple times with ultrapure water. Finally, the activated adsorbent was freeze-dried and reused in the next sorption cycle.

## 3. Results and discussion

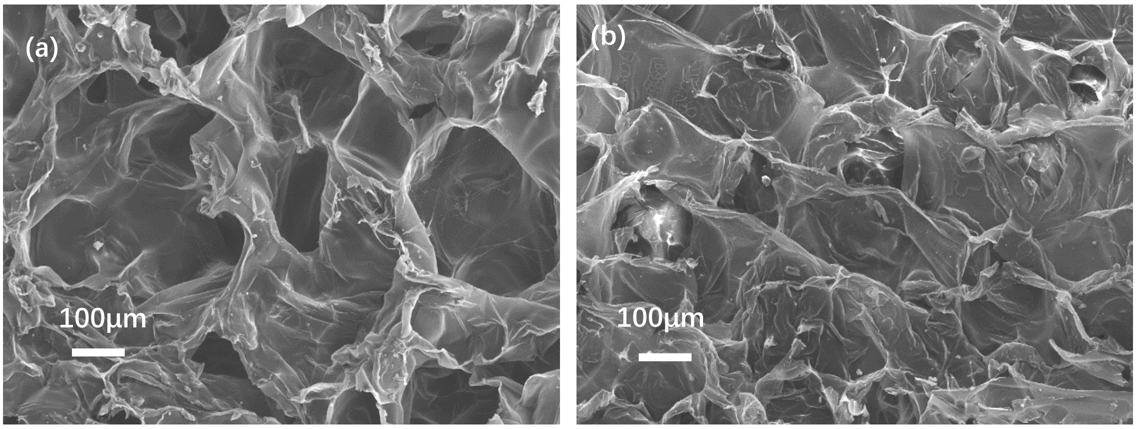


**Figure S1.** The SEM image of CSA (left) and CSPA (right).

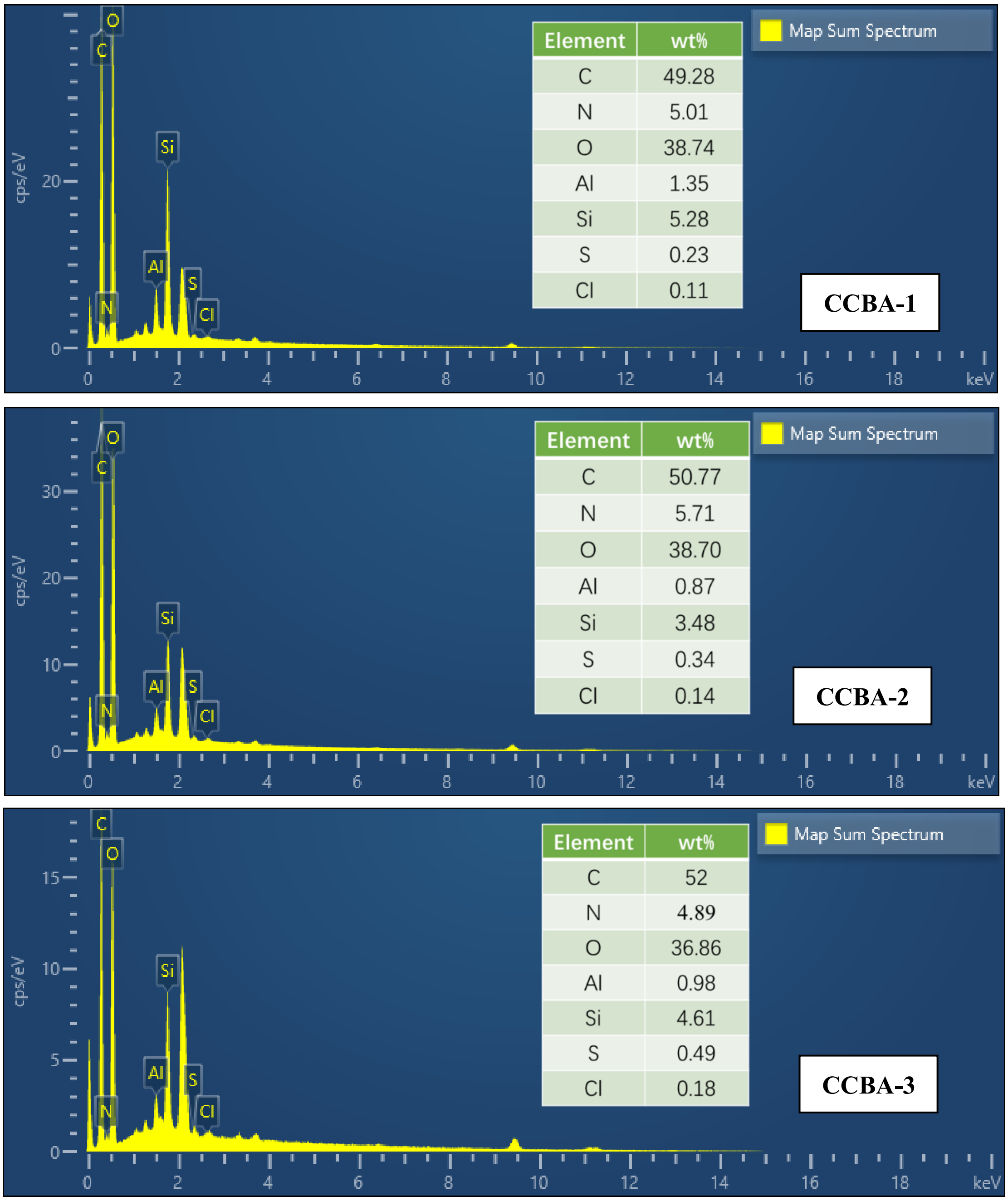

**Figure S2.** The EDS spectrum of CCBA-1, CCBA-2 and CCBA-3.

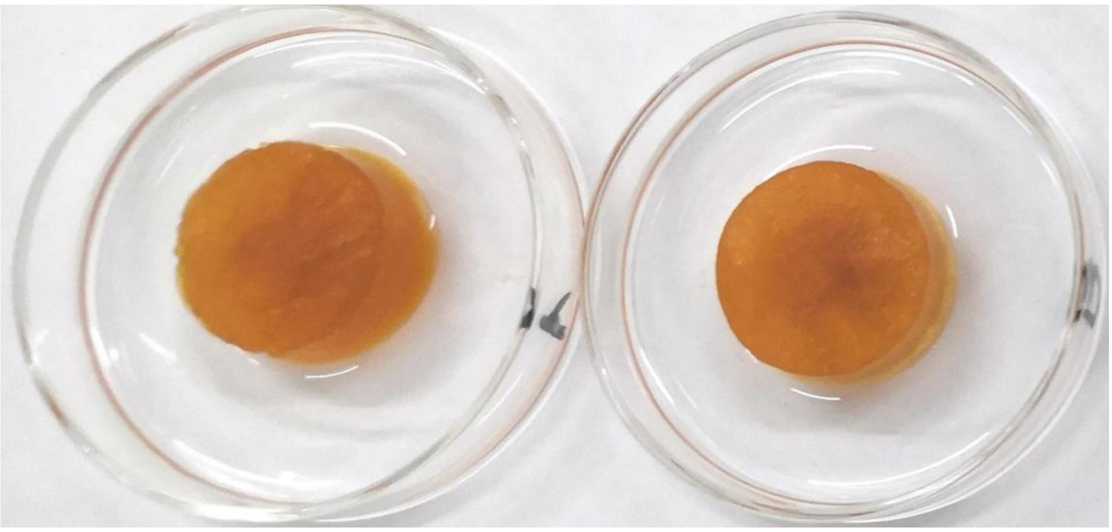

**Figure S3.** Digital graph of CSA (left), CSPA (right) before and after loaded same weight (500g) under water saturated condition.

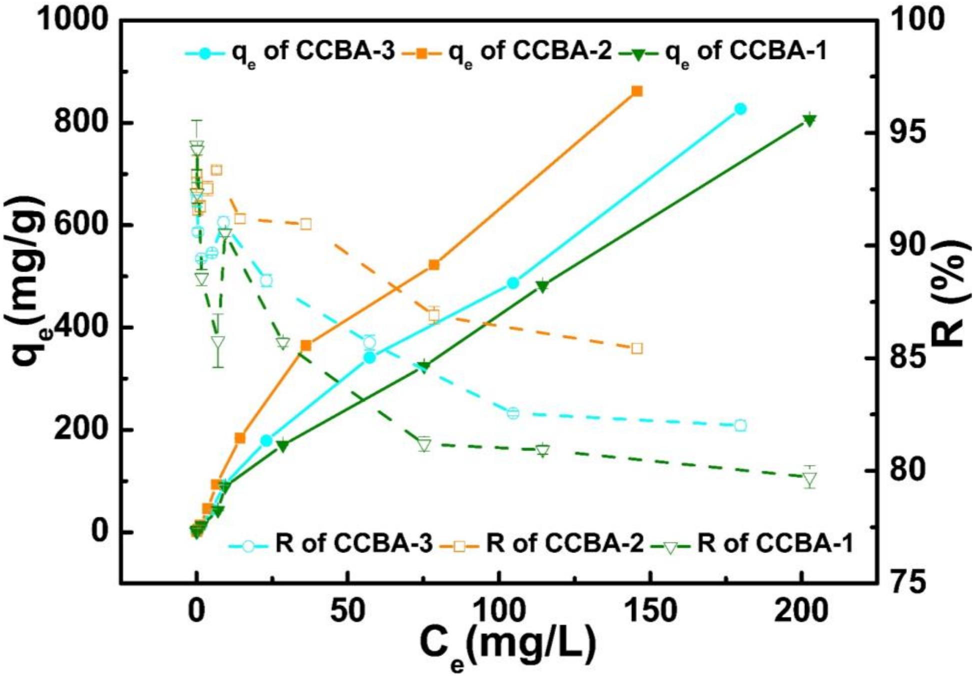


**Figure S4.** The effect of initial concentration on adsorption capacity of CCBA to Cu (II). ($C_0$=1-1000mg/L, t=240min, m=20mg, V=20mL, pH=5.5, T=303K)

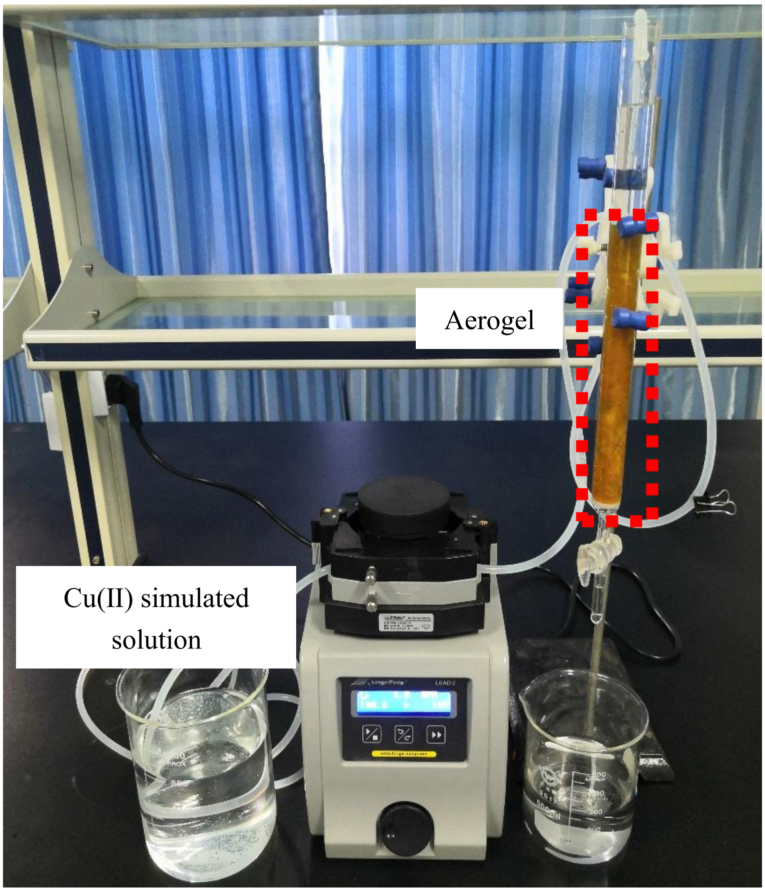

**Figure S5.** The digital image of self-fabricated fixed-bed column.

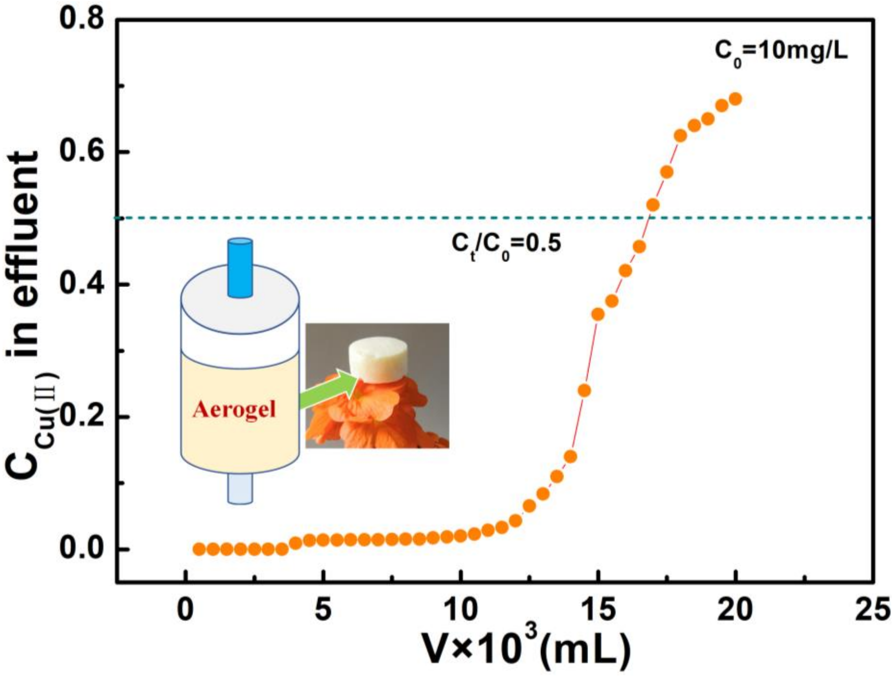


**Figure S6.** Breakthrough curve of $Cu^{2+}$ on CCBA-2.

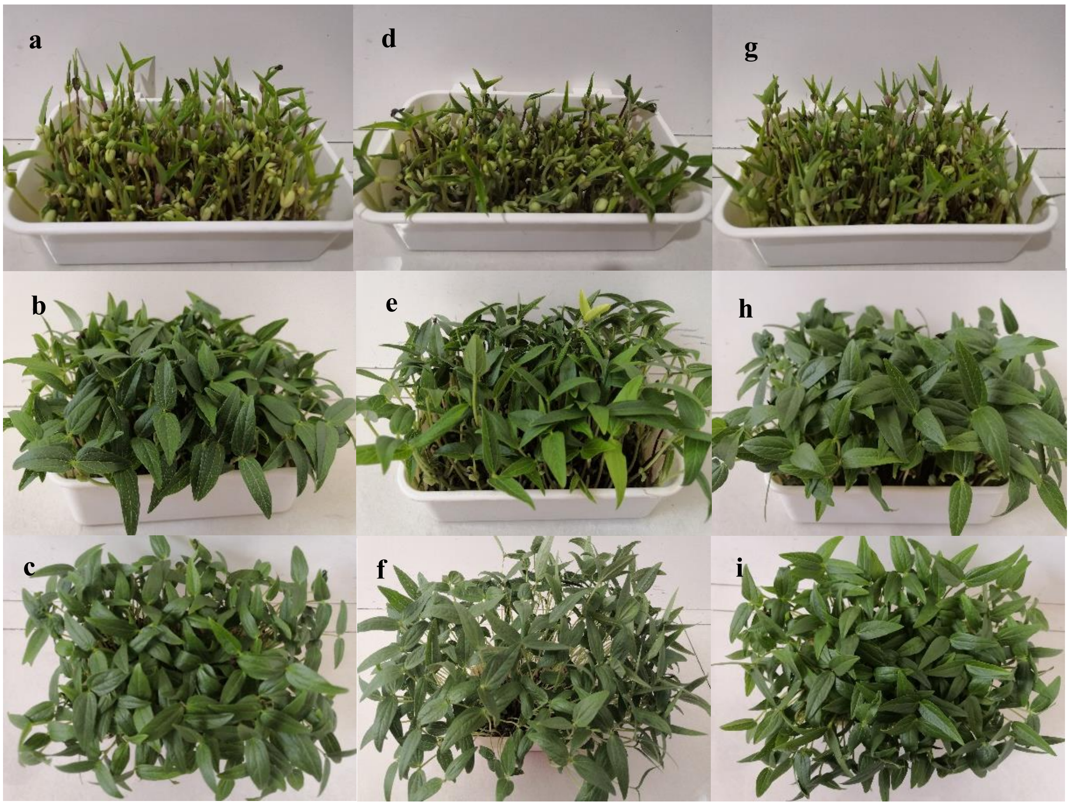

**Figure S7.** Growth status of mung bean sprout cultivated with pure tap water (a-c), tap water (10 mg/L) (d-f) and effluent of tap water after fixed bed column experiment (g-i).

According to Figure S7, the growth of mung beans cultivated in the effluent (S8g-i) from the fixed-bed column was similar to that in the blank group (S8a-c), except for a noticeable decrease in the germination rate of mung beans cultured in untreated tap water (S8d-f). This suggests that the ecological risk of using the effluent for farmland irrigation is acceptable. It is feasible to use the effluent, treated by the CCBA-2 packed fixed-bed column, for irrigating farmland with low concentrations of copper-containing solution. In summary, the comprehensive utilization of the effluent after column adsorption shows potential for agricultural irrigation.

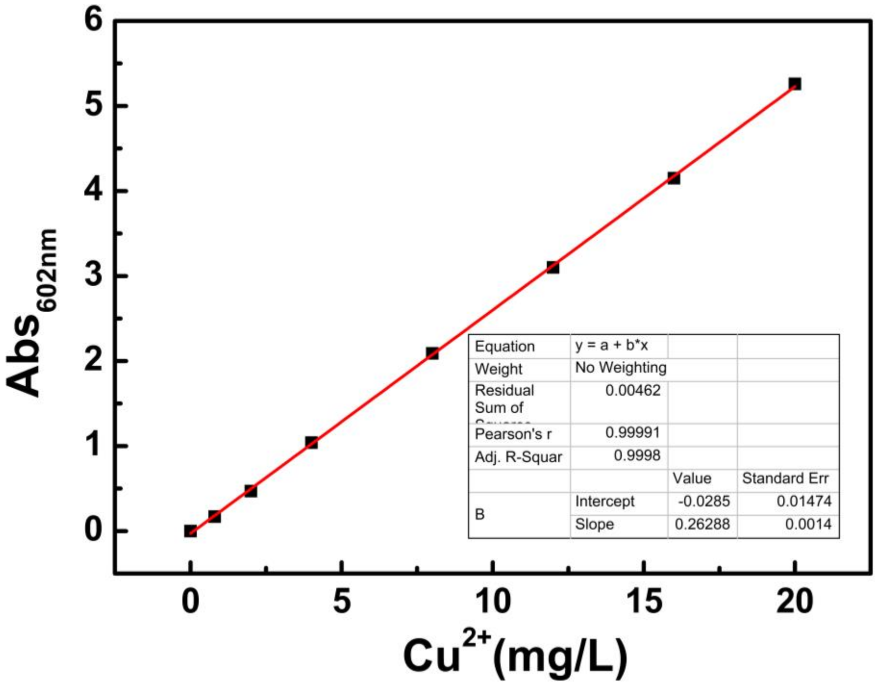


**Figure S8.** Calibration curve determined at 25 ℃ for $Cu^{2+}$ dissolved in Ultrapure water, pH 5.5.

# Tables

| Materials | BET surface aera ($m^2 \cdot g^{-1}$) | Average pore size (nm) | Pore volume ($cm^3 \cdot g^{-1}$) |
|---|---|---|---|
| CCBA-1 | 41.28 | 15.25 | 0.024 |
| CCBA-2 | 48.36 | 6.52 | 0.008 |
| CCBA-3 | 46.57 | 7.71 | 0.010 |

**Table S1.** The BET surface area, average pore size and pore volume of CCBA-1, CCBA-2 and CCBA-3.

| Component | Tap water | Ili River water | Actual copper-containing wastewater |
|---|---|---|---|
| pH | 7.65 | 7.67 | 3.28 |
| TN (mg/L) | 0.21 | 1.03 | 12.72 |
| $NH_3$-N (mg/L) | 0.24 | 0.48 | 8.75 |
| $SO_4^{2-}$ (mg/L) | 26.42 | 47.7 | 58.3 |
| $Cu^{2+}$ (mg/L) | 383.4 | 373.27 | 386.28 |
| $Cl^-$ (mg/L) | 0.33 | 151 | 157.4 |
| TP (mg/L) | 0.05 | 0.22 | 4.23 |
| COD (mg/L) | 4.4 | 19.4 | 146 |

**Table S2.** The original indicators of tap water, Ili River water and actual copper-containing wastewater.

| Parameter | m/g | D/cm | H/cm | $Q_v$/mL·min$^{-1}$ | t (min) |
|---|---|---|---|---|---|
| column | 3 | 2 | 20 | 2 | 31.4 |

m: the total mass of the BCCA-2 in the column; D: inner diameter of column; H: column depth; $Q_v$: volumetric flow rate; t: the hydraulic retention time; Temperature: 25-28℃.

**Table S3.** Fixed-bed column parameters.

# Videos

Video 1.mp4

**Video 1**. CCBA before and after compression under water saturated condition.

Video 2.mp4

**Video 2.** Hydrophilicity test of CCBA-1.
